# Phenylbutyrate restricts murine β-coronavirus infectivity and limits virus-induced demyelination *in vivo*

**DOI:** 10.1128/jvi.00819-25

**Published:** 2025-09-08

**Authors:** Grishma Kasle, Madhav Sharma, Saurav Kumar, Pranati Das, Subhajit Das Sarma, Michael Koval, Lawrence C. Kenyon, Jayasri Das Sarma

**Affiliations:** 1 Department of Biological Sciences, Indian Institute of Science Education and Research Kolkata 99007, Mohanpur, West Bengal, India; 2 BioInspired Innovations Pvt. Ltd, Kolkata, West Bengal, India; 3 Small Animal Facility, Indian Institute of Science Education and Research Kolkata 99007, Mohanpur, West Bengal, India; 4 Division of Pulmonary, Allergy, Critical Care and Sleep Medicine, Department of Medicine, Emory University School of Medicine 728540 , Atlanta, Georgia, USA; 5 Department of Cell Biology, Emory University School of Medicine 12239, Atlanta, Georgia, USA; 6 Department of Pathology, Cooper University Health Care 686126, Camden, New Jersey, USA; 7 Department of Ophthalmology, University of Pennsylvania 43946, Philadelphia, Pennsylvania, USA; The Ohio State University, Columbus, Ohio, USA

**Keywords:** β-coronavirus, mouse hepatitis virus, gap junction intercellular communication, connexin43, connexin47, ERp29, 4-PBA, multiple sclerosis

## Abstract

High morbidity and mortality associated with human β-coronavirus (CoV) infection highlight the need to determine host responses to infection and develop anti-viral therapies. Gap junction intercellular communication (GJIC), particularly involving Connexin43 (Cx43), is vital for maintaining central nervous system (CNS) homeostasis, and disruption of GJIC is a well-documented pathogenic mechanism among β-coronaviruses. Specifically, murine β-coronavirus, mouse hepatitis virus (MHV-A59) inoculation in the mouse brain causes acute-stage CNS viral spread and chronic neuroinflammatory demyelination while causing pronounced downregulation of Cx43 at the acute stage, reflecting a critical role in CNS pathology. To pharmacologically target Cx43, 4-phenylbutyric acid (4-PBA) has been widely used, since it enhances the expression of ER-resident thioredoxin family protein (ERp29), a molecular chaperone of Cx43. 4-PBA has been shown to have anti-viral effects against MHV-A59 *in vitro*; however, the *in vivo* efficacy of 4-PBA is unknown. This study investigates the effect of 4-PBA administration on the pathology of MHV-A59 infection *in vivo*. We demonstrate that 4-PBA treatment reduced acute MHV-A59 infectivity and viral spread in the brain while modulating the glial cell response and mounting host immunity. MHV-A59 infection downregulated the expression of ERp29 in the CNS, which was rescued by 4-PBA treatment. Furthermore, treatment with 4-PBA effectively preserved the expression of Cx43 and Cx47 in infected CNS cells, counteracting their infection-induced downregulation. 4-PBA treatment not only limits acute viral replication and spread throughout the brain but also protects against severe chronic virus-induced neuroinflammatory demyelination *in vivo*. These findings highlight 4-PBA’s significant anti-viral potential against murine β-CoV and therapeutic potential in mitigating virus-induced neuroinflammatory demyelination.

**IMPORTANCE:**

Past outbreaks and the emergence of novel coronaviruses pose a serious global health threat, warranting studies on the disease mechanism of these viruses and the development of new anti-viral strategies. In the current study, we demonstrated the antiviral potential of 4-phenylbutyric acid (4-PBA) against a neurotropic murine β-coronavirus, mouse hepatitis virus (MHV-A59). MHV-A59 inoculation in the brain causes virus infection and disruption of gap junction (GJ) communication by the downregulation of GJ proteins connexin 43 (Cx43) and connexin 47 (Cx47), crucial for maintaining CNS homeostasis. We demonstrate that 4-PBA restricts viral spread and infectivity in the mouse brain and improves the reduced levels of ERp29 and, thus, Cx43 and Cx47 in the infected CNS. Furthermore, 4-PBA mitigated virus-induced chronic neuroinflammatory demyelination, the characteristic feature of multiple sclerosis (MS). These findings demonstrate that 4-PBA holds significant therapeutic potential for restricting β-CoV spread and virus-induced neuroinflammatory demyelination.

## INTRODUCTION

β-coronaviruses (CoV), including severe acute respiratory syndrome CoV-1 (SARS-CoV-1), Middle East respiratory syndrome CoV (MERS-CoV), and severe acute respiratory syndrome CoV-2 (SARS-CoV-2), have posed significant threats to human health. The highly pathogenic and contagious nature of these human coronaviruses makes experimental animal models indispensable for studying their pathogenic mechanisms and testing potential antiviral strategies. Murine β-coronaviruses (CoV), such as the hepato-neurotropic strain MHV-A59, have been used to gain insight into the mechanisms of virus-induced CNS demyelination (1). Recent reports highlighting CNS manifestations of β-CoVs underscore the importance of this model in gaining insights into the CNS pathogenesis of CoVs (2, 3).

Intracranial inoculation of MHV-A59 in C57BL/6 mice causes acute infection and neuroinflammation in the CNS at day 5 post-infection (p.i), followed by chronic (day 30 p.i) inflammatory demyelination. This mimics pathological features of human MS, making it an important animal model for studying virus-induced demyelination and MS pathogenesis (1). This makes this model unique in not only gaining insight into the pathogenesis of CoVs but also elucidating the cellular and molecular mechanisms of virus-induced demyelination in MS.

β-CoVs, including MHV-A59, OC43, and SARS-CoV-2, are known to alter gap junction intercellular communication (GJIC) (4–9). In the CNS, GJIC is crucial to forming pan-glial networks, enabling metabolic coupling and maintaining homeostasis. These gap junctions (GJs) are made up of connexin proteins. Connexins assemble into hexameric hemichannels or connexons, and when connexons of juxtaposed cells dock together, they form a GJ. This enables direct cell-cell communication through GJ channels that interconnect the cytoplasm of adjacent cells (10–15). GJs enable the passive intercellular diffusion of small molecules, including glutamate, glutathione, glucose, adenosine triphosphate (ATP), cyclic adenosine monophosphate (cAMP), inositol 1,4,5-trisphosphate (IP3), and ions (Ca2^+^, Na^+^, and K^+^). Various glial cell types express different connexins. Astrocytes express multiple different connexins (Cx43, Cx30, and Cx26), as do oligodendrocytes (Cx32, Cx29, and Cx47), microglia (Cx43, Cx32, and Cx36), and endothelial cells (Cx37, Cx40, and Cx43) (4, 10). Connexin43 (Cx43) is the most highly expressed and abundant in the brain because it is involved in extensive GJ coupling between astrocytes, the most abundant CNS cell type.

MHV-A59 infection in the CNS leads to the downregulation of Cx43 expression at the acute stage, which causes destabilization and downregulation of its coupling partner, Cx47, on the oligodendrocyte (5, 6). MHV-A59 infection and inflammation-mediated alterations in Cx43 and Cx47 expression and their effect on heterotypic GJIC formation between astrocytes and oligodendrocytes are likely to be associated with the observed chronic demyelination in this model.

*In vitro* studies of exogenous ER-resident thioredoxin family chaperone protein (ERp29) overexpressing MHV-A59-infected cells have shown that ERp29 regulates Cx43 trafficking to the cell surface (7, 16, 17). However, it remains to be elucidated if ERp29 expression is altered in the CNS during MHV-A59 infection *in vivo* and if that correlates with changes in the expression of Cx43 in the infected CNS. Moreover, ERp29 expression and connexin assembly into gap junctions can be pharmacologically manipulated *in vitro using* 4-phenylbutyric acid (4-PBA), an agent that is FDA-approved for treating urea cycle disorders and is under investigation in several clinical trials (18–20). Previous studies have demonstrated the potential of 4-PBA to improve Cx43 trafficking to the cell surface, which would otherwise be retained in the intracellular compartment (7, 17, 21). *In vitro* studies of MHV-A59-infected glial cells have established that 4-PBA lessens the severity of infection by rescuing Cx43 trafficking due to increased ERp29 expression (7). Whether 4-PBA is effective in reducing CNS pathology due to MHV-A59 infection has not been examined *in vivo*.

Here, we found that 4-PBA treatment reduced *in vitro* and *in vivo* acute-stage MHV-A59 infectivity and spread. Furthermore, 4-PBA treatment in mice modulated the glial cell response to infection. We demonstrate that overall ERp29 expression is downregulated in the infected CNS during the acute stage, with a marked loss in MHV-A59-infected cells. In contrast, 4-PBA-treated mice preserved ERp29 expression, even in infected cells. There was a parallel downregulation in Cx43 and Cx47 expression in the infected CNS, which was mitigated by 4-PBA treatment. Furthermore, 4-PBA treatment was able to protect mice against chronic-stage virus-induced demyelination. The present study provides strong evidence to support the anti-viral efficacy of 4-PBA against murine β-coronavirus and proposes to explore its potential in mitigating human coronaviruses-associated pathology.

## RESULTS

### 4-PBA treatment improved Cx43 trafficking to the cell surface and reduced MHV-A59 infectivity in mouse primary astrocytes

We previously demonstrated in the astrocytoma-derived cell line, DBT cells, that 4-PBA has the ability to upregulate ERp29, enhance Cx43 trafficking, and reduce the severity of MHV-A59 infectivity and spread (7, 16, 17). Therefore, we first examined whether 4-PBA treatment had a comparable effect on MHV-A59-infected mouse primary astrocytes *in vitro*.

**Fig 1:**
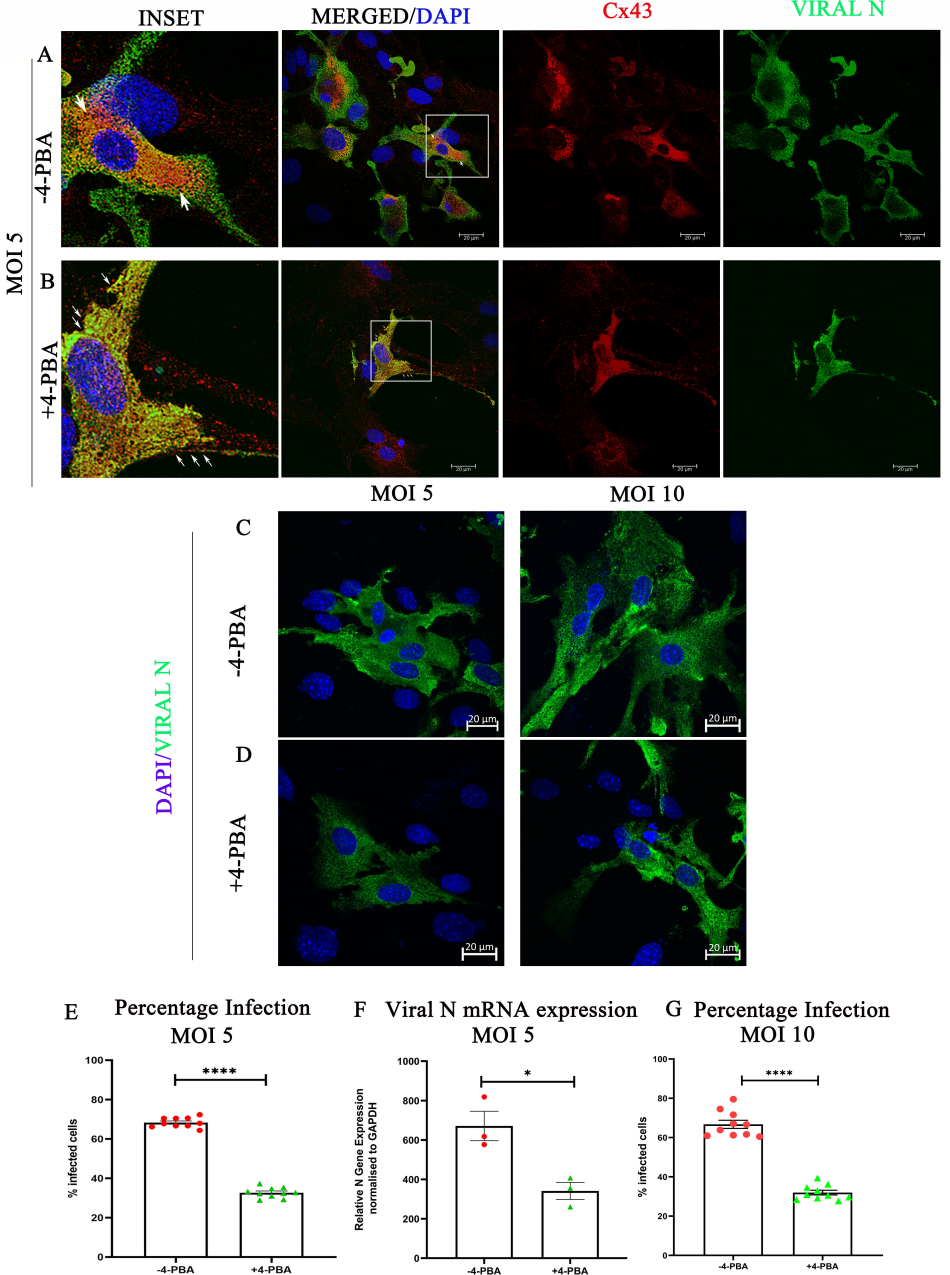
4-PBA treatment improves Cx43 trafficking to the cell surface and reduces MHV-A59 infectivity in mouse primary astrocytes. (**A and B**) Representative confocal photomicrographs of murine primary astrocytes infected with MHV-A59 at MOI 5 in either the presence (**B**) or absence (**A**) of 4-PBA for 24 h p.i., analyzed by immunolabeling with anti-Cx43 (red) and anti-nucleocapsid (Viral-N) antibody (green), and mounted in a DAPI-containing mounting medium. The boxed areas are shown alongside as manually zoomed insets. Arrows indicate intracellular retention of Cx43 in (**A**) and Cx43 puncta in (B) in infected cells. (**C and D**) Representative confocal photomicrographs of murine primary astrocytes infected with MHV-A59 at MOI 5 and MOI 10, respectively, in either the presence (**D**) or absence (**C**) of 4-PBA for 24 h p.i., analyzed by immunolabeling with anti-nucleocapsid (Viral-N) antibody (green) and mounted in a DAPI-containing mounting medium. (**E**) Quantification of percentage infected cells in untreated (−4-PBA) or 4-PBA treated (+4-PBA) astrocyte cultures infected at MOI 5. (**F**) shows the differential viral mRNA expression in untreated or 4-PBA-treated astrocyte cultures infected with MHV-A59 at MOI 5. (**G**) Quantification of percentage infected cells in untreated (−4-PBA) or 4-PBA treated (+4-PBA) astrocyte cultures infected at MOI 10. The results were expressed as mean ± SEM (*n* = 3 per group). Asterisks represent statistical significance calculated using an unpaired Student’s *t*-test and Welch correction; P < 0.05 was considered significant. *P< 0.05, ****P < 0.0001.

Primary murine astrocytes were isolated from neonatal mouse pups, infected with MHV-A59 at a multiplicity of infection (MOI) of 5, either untreated (−4-PBA) or treated (+4-PBA) with 4-PBA for 24 h. Immunofluorescence studies demonstrated that in MHV-A59-infected cells, astrocytes exhibit intracellular retention of Cx43 (Fig. 1A, insets, retention indicated by arrows). Although 4-PBA treatment in MHV-A59-infected astrocytes restored the ability of Cx43 to form puncta at the cell surface (Fig. 1B, Insets, puncta indicated by arrows), indicating improved trafficking of Cx43, which was consistent with our previous reports demonstrating that 4-PBA has the ability to promote Cx43 trafficking (7, 16, 17).

Furthermore, the percentage of infected cells was lower in cells that received 4-PBA treatment (Fig. 1D, E, and G) compared with those that did not receive any treatment (Fig. 1C, E, and G) at both MOI 5 (Fig. 1C through E) and MOI 10 (Fig. 1C, D, and G). Similarly, viral N gene RNA transcript levels were significantly lower in 4-PBA-treated cultures compared with those that did not receive any treatment (Fig. 1F). Thus, 4-PBA reduces MHV-A59 viral infectivity of primary astrocytes *in vitro*. This finding further supported studying the efficacy of 4-PBA treatment against MHV-A59 infection *in vivo*.

### 4-PBA treatment restricts MHV-A59 infection and spread in the brain at the acute stage of infection

The MHV-A59-induced disease in 4-week-old C57BL/6 mice manifests into two temporally distinct stages: acute and chronic. Upon intracranial inoculation with the virus, the disease initially presents with robust viral replication and neuroinflammation in the CNS, accompanied by hepatitis. This acute stage of infection peaks at day 5 p.i. After day 5 p.i., the viral titer gradually decreases, resolving the neuroinflammation by day 10 p.i., although the viral RNA persists at low levels. Myelin loss, with or without axonal loss, starts as early as day 7 p.i. and reaches its peak at the chronic stage of infection at 30 days p.i. This pattern of neurodegeneration mimics certain pathological features of the human neurological disease MS (1). Thus, the current study is focused on these two critical time points: the acute stage at day 5 p.i. and the chronic stage at day 30 p.i.

**Fig 2:**
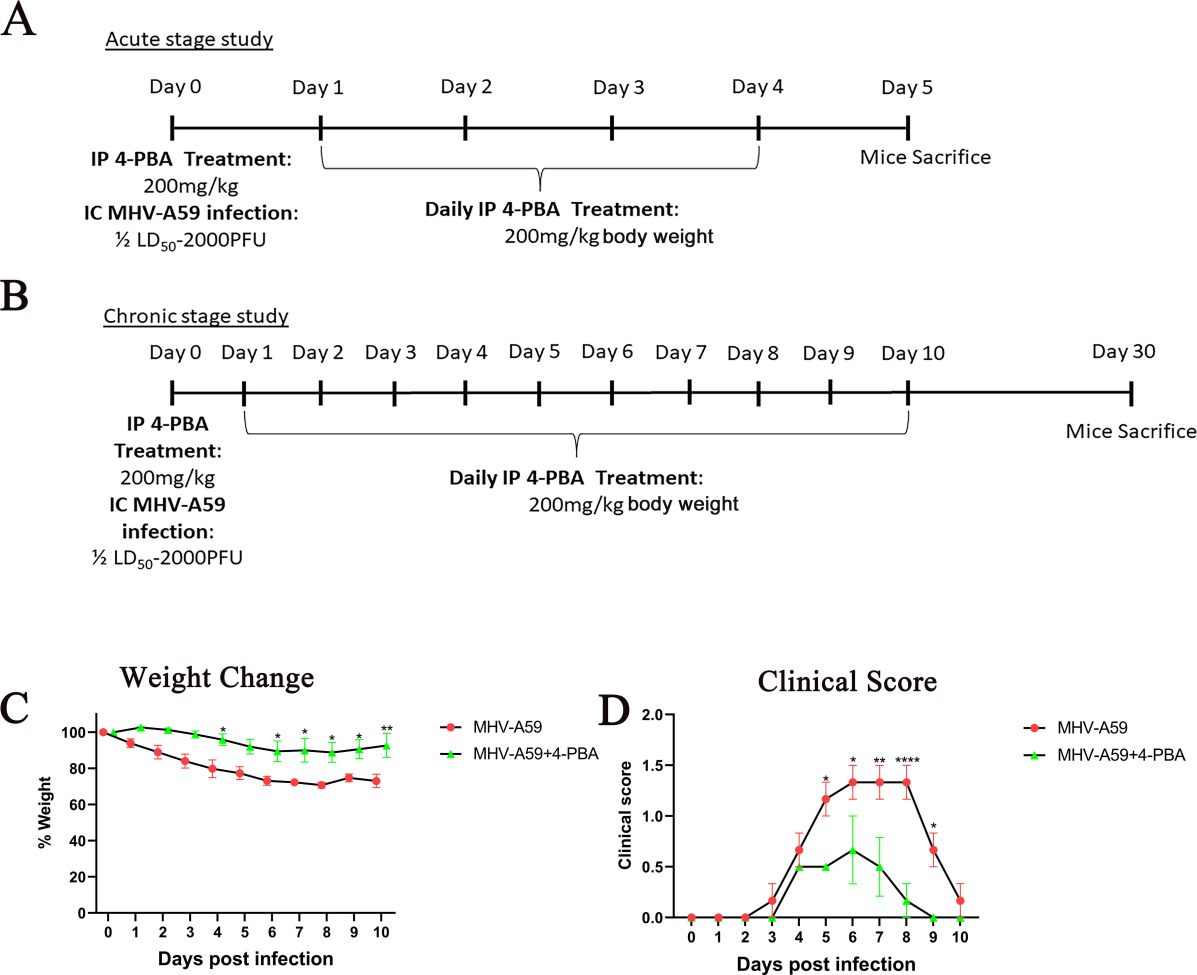
4-PBA treatment improves the weight loss and disease severity score in MHV-A59-infected mice. (**A and B**) Schematic representation of the treatment and infection design. Treated C57BL/6 mice received intraperitoneal injections of 4-PBA (200 mg/kg) followed by intracranial inoculation of MHV-A59 (2000 PFU) on day 0 for both acute (**A**) and chronic stage studies (**B**). Day 1 onward, mice were administered 4-PBA daily up to day 4 for the acute stage study (**A**) and up to day 10 for the chronic stage study (B). Non-treated and mock-infected mice were maintained as controls. Mice infected with MHV-A59 that received 4-PBA treatment (MHV-A59+4-PBA) or did not receive any treatment (MHV-A59) were monitored daily for (**C**) weight change and (**D**) development of disease signs and symptoms. Disease severity scores were assigned a relative scale of 0–4 as described in Materials and Methods. All mice demonstrated 100% survival in both treated and untreated groups. The results were expressed as mean ± SEM from two independent biological experiments (*n* = 6 per group). Asterisks represent statistical significance calculated using two-way ANOVA. *P* < 0.05 was considered significant. *P < 0.05, **P < 0.01, ****P < 0.0001.

Mice were divided into two groups: one group (MHV-A59) received intracranial (IC) inoculation with MHV-A59 (50% of the LD50 dose: 2000 PFU) on day 0. The second group (MHV-A59+4-PBA) was infected with the same dose of MHV-A59 and received an intraperitoneal (IP) injection of 4-PBA at 200 mg/kg of body weight 0.5 h prior to IC inoculation. Additionally, the MHV-A59+4-PBA group received daily 4-PBA injections of 200 mg/kg of body weight/day until day 4 for acute stage studies and until day 10 for chronic stage studies (Fig. 2A and B). Vehicle control mice (that received IP PBS injection) were also produced and maintained in parallel.

Mice were monitored daily for weight change and the development of disease signs and symptoms. We observed that MHV-A59+4-PBA-treated mice showed less than 10% weight loss, whereas MHV-A59-infected mice lost almost 20% of their original weight by day 4 p.i. (Fig. 2C). MHV-A59-infected mice showed a progressive increase in disease severity score starting day 3 p.i., reaching an average score of 1–1.5 by day 5–8 as indicated by a hunched back phenotype with the occasional presence of mild ataxia and hind-limb weakness. In contrast, MHV-A59+4- PBA-treated mice maintained a lower disease severity score with a maximum average of 0.5, indicative of ruffled fur and possibly slower movement (Fig. 2D).

**Fig 3:**
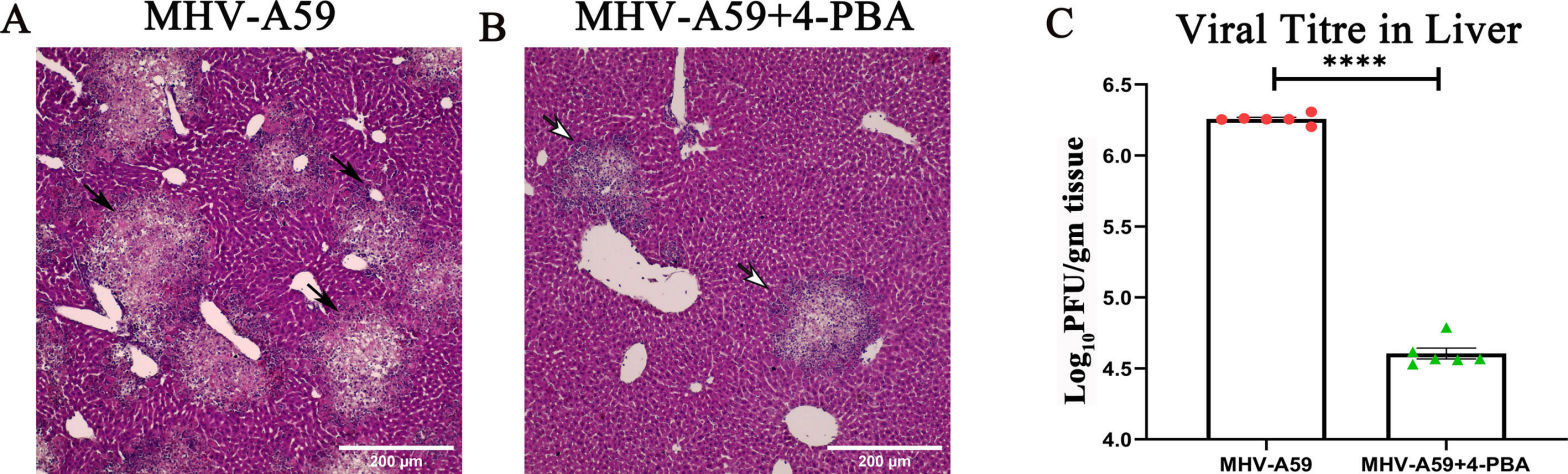
4-PBA treatment reduces acute hepatitis upon MHV-A59 infection. MHV-A59 infection is known to cause acute mild to moderate hepatitis. On day 5 p.i., MHV-A59-infected (**A**) and MHV-A59+4-PBA (**B**) treated mice were subjected to histopathological analyses of liver tissues by H & E staining. Arrows indicate inflamed and necrotic hepatic lesions. (**C**) Viral titer was determined in liver homogenates at day 5 p.i. and plotted. The results were expressed as mean ± SEM from two independent biological experiments (*n* = 6 per group). Asterisks represent statistical significance calculated using an unpaired Student’s *t*-test and Welch correction; P < 0.05 was considered significant. ****< 0.0001. Scale bar 200 µm.

MHV-A59 is a dual hepato-neurotropic virus in which liver inflammation is a valuable indicator of disease severity and may contribute to overall pathology. Acute mild-to-moderate hepatitis at day 5 p.i. is characteristic of MHV-A59 infection and was observed in infected mice with the presence of multiple necrotizing hepatic lesions spread throughout the liver (Fig. 3A). However, MHV-A59+4-PBA-treated mice showed fewer hepatic lesions, indicative of decreased liver inflammation (Fig. 3B). Furthermore, viral titers in the liver of MHV-A59+4-PBA-treated mice were significantly lower compared with MHV-A59-infected mice (Fig. 3C).

**Fig 4:**
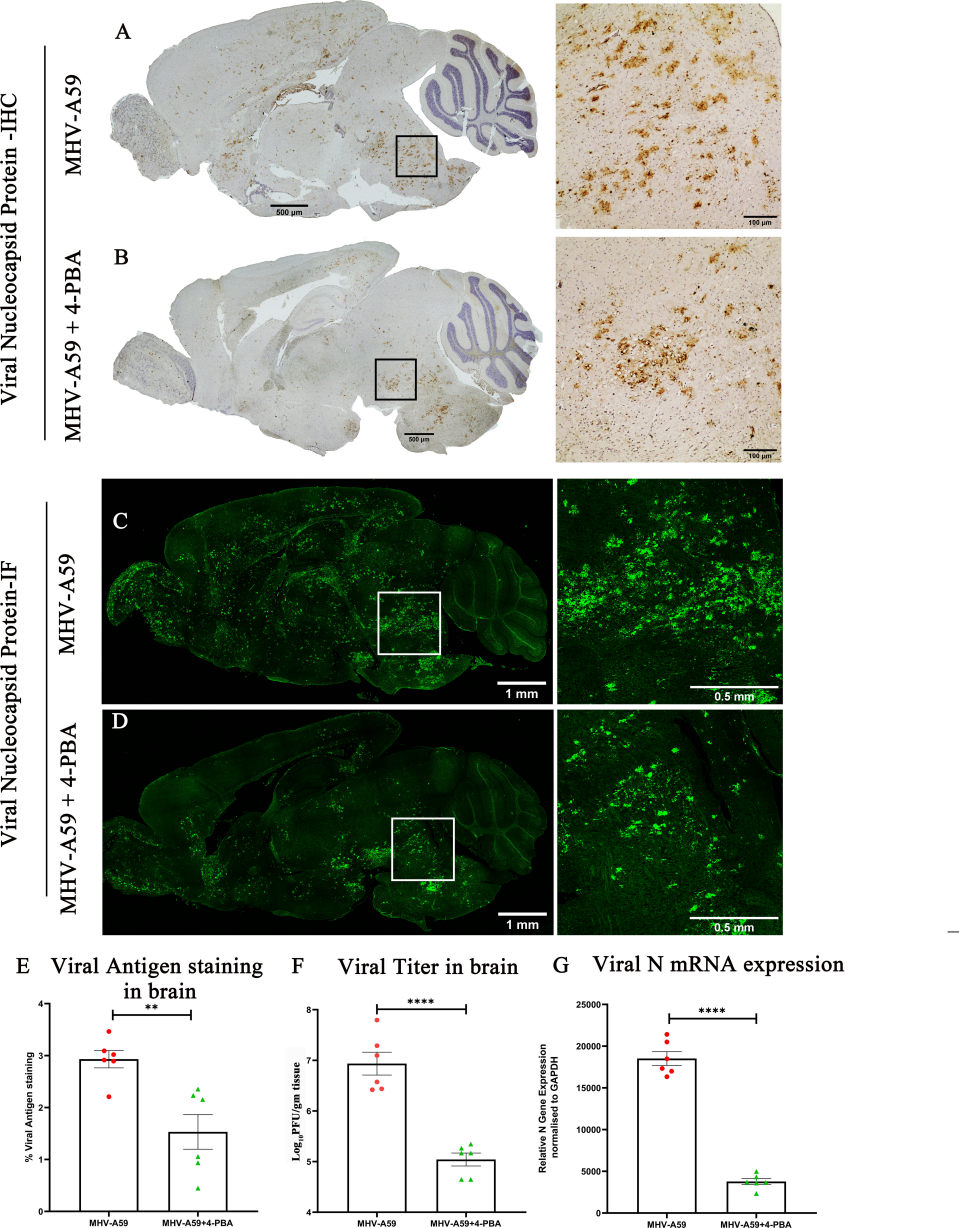
4-PBA treatment restricts *in vivo* spread and infectivity of MHV-A59 in the mouse brain. (**A and B**) On day 5 p.i., serial mid-sagittal brain sections (5 µm thick) from MHV-A59 (**A**) and MHV-A59+4-PBA (**B**) sets of mice were immunohistochemically (IHC) stained with anti-viral nucleocapsid (anti-N) antibody. The boxed areas are shown at higher magnification alongside the corresponding brain midsagittal sections. (**C and D**) Photomicrographs of serial mid-sagittal brain sections (5-µm thick) from MHV-A59 (**C**) and MHV-A59+4-PBA (**D**) sets of mice stained by immunolabeling with anti-N antibody and visualized using fluorescence. The boxed areas are shown at higher magnification alongside the corresponding brain midsagittal sections. (**E**) Quantification of Viral N antigen staining by IHC (**A and B**) in the brain. (**F**) Comparative viral titer and (**G**) differential viral mRNA expression in the brain. The results were expressed as mean ± SEM from two independent biological experiments (*n* = 6 per group). Asterisks represent statistical significance calculated using an unpaired Student’s *t*-test and Welch correction. *P* < 0.05 was considered significant. **P < 0.01,****P < 0.0001.

To confirm if the reduced disease severity in MHV-A59+4-PBA-treated mice correlated with reduced viral spread and infection in the brains of treated mice, we further estimated the viral spread and titers in the brains of experimental mice. Serial paraffin-embedded brain sections from both MHV-A59-infected and MHV-A59+4-PBA-treated mice were immunohistochemically stained with anti-viral nucleocapsid antibodies. In correlation with the reduced disease severity, the viral antigen staining was significantly lower in MHV-A59+4-PBA-treated mice (1.531% area of staining) as opposed to MHV-A59 (2.932% area of staining) infected mice without 4-PBA treatment (Fig. 4A, B, and E). Immunofluorescence further confirmed that 4-PBA treatment reduced viral spread, including reduced viral antigen staining in the brain (Fig. 4C and D).

To assess if the reduced disease severity and viral antigen staining correlated with reduced viral replication and infectious viral titers, the brains of MHV-A59 and MHV-A59+4-PBA mice were assayed by plaque assay. Consistent with a reduction in viral antigen spread in response to 4-PBA treatment, brain tissue from MHV-A59+4-PBA-treated mice had fewer infectious viral particles than brain tissue from MHV-A59-infected mice (Fig. 4F). Finally, we measured the effect of 4-PBA on viral mRNA. There were fewer viral nucleocapsid transcripts found in MHV-A59+4-PBA-treated brains compared with MHV-A59-infected brains, which correlated with levels of viral antigen and production of infectious viral particles (Fig. 4G).

Taken together, these results demonstrate the significant *in vivo* antiviral efficacy of 4-PBA against MHV-A59 infection in mice, effectively reducing disease severity, including weight loss, hepatitis, viral titers in both the brain and the liver, as well as viral spread in the brain.

### 4-PBA-modulated glial cell activation and inflammation upon MHV-A59 infection

With reduced viral spread upon 4-PBA treatment, we anticipated a difference in neuroinflammation in response to MHV-A59 infection and the activation of glial cells that are critical to neuroinflammation. To investigate this, we assessed the activation pattern of microglia and astrocytes in paraffin-embedded brain sections from MHV-A59 and MHV-A59+4-PBA mice.

**Fig 5:**
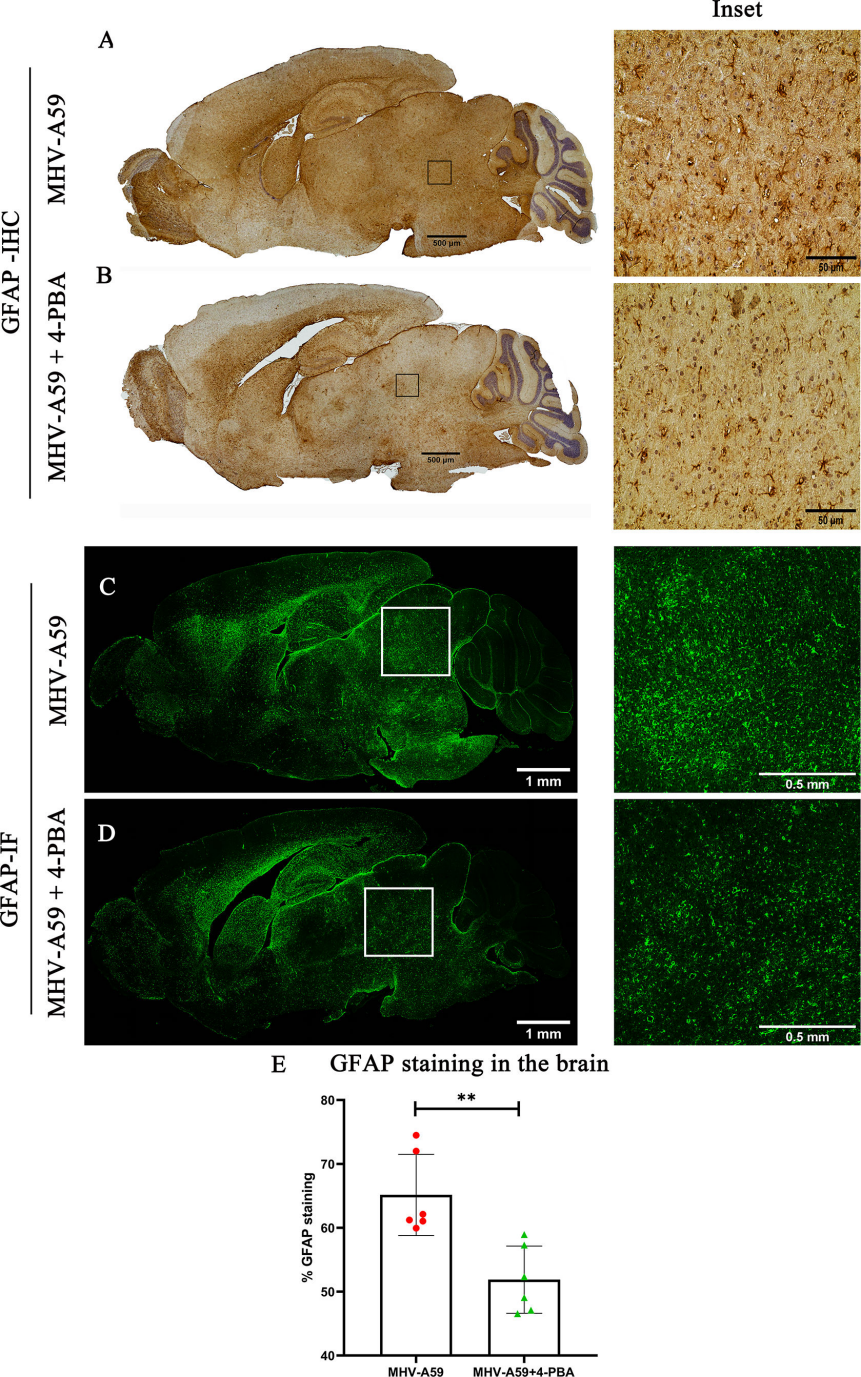
4-PBA treatment reduces astrocyte activation upon MHV-A59 infection in the mouse brain. (**A and B**) On day 5 p.i., serial mid-sagittal brain sections (5 µm thick) from MHV-A59 (**A**) and MHV-A59+4-PBA (**B**) treated mice were immunohistochemically (IHC) stained with anti-GFAP antibody. The boxed areas are shown at higher magnification alongside the corresponding brain midsagittal sections. (**C and D**) Photomicrographs of serial mid-sagittal brain sections (5 µm thick) from MHV-A59 (**C**) and MHV-A59+4-PBA (**D**) mice stained by immunofluorescence with anti-GFAP antibody. The boxed areas are shown at higher magnification alongside the corresponding brain midsagittal sections. (**E**) Quantification of GFAP antigen staining by IHC in the brain. Results were expressed as mean ± SEM from 2 independent biological experiments (*n* = 6 per group). Asterisks represent statistical significance calculated using unpaired Student’s *t*-test and Welch correction. *P* < 0.05 was considered significant, **P < 0.01.

Astrocyte activation was determined in serial brain sections using immunohistochemical staining for GFAP (glial fibrillary acidic protein), the most widely used marker of reactive astrocytes (Fig. 5A and B). The quantification of GFAP staining intensity revealed significantly lower immunoreactivity in MHV-A59+4-PBA mice, consistent with lower viral load compared with MHV-A59-infected mice (Fig. 5E). Immunofluorescence analysis further confirmed there was an overall reduction in GFAP staining/astrocyte activation in MHV-A59+4-PBA mice (Fig. 5C and D).

**Fig 6:**
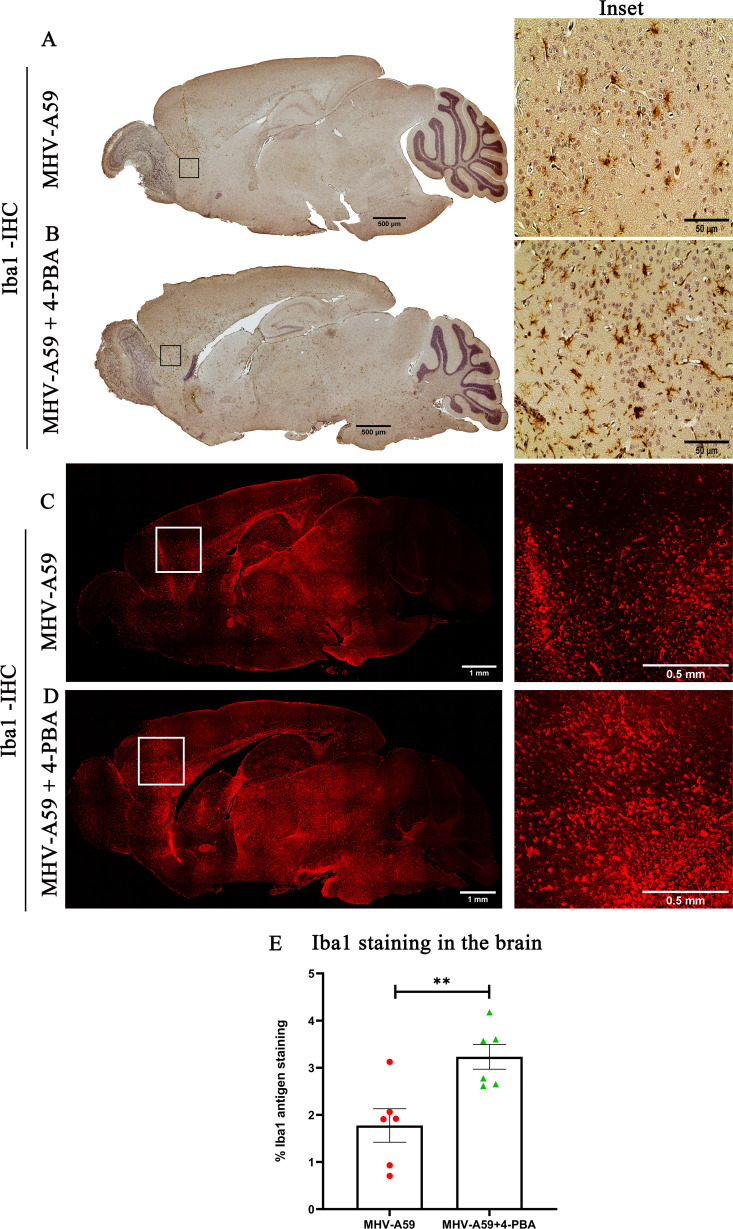
Iba1^+^ microglia/macrophage activation in response to MHV-A59 infection is enhanced upon 4-PBA treatment. (**A and B**) On day 5 p.i., serial mid-sagittal brain sections (5 µm thick) from MHV-A59 (**A**) and MHV-A59+4-PBA (**B**) mice were immunohistochemically (IHC) stained with anti-Iba1 antibody. The boxed areas are shown at higher magnification alongside the corresponding brain midsagittal sections. (**C and D**) Photomicrographs of serial mid-sagittal brain sections (5 µm thick) from MHV-A59 (**C**) and MHV-A59+4-PBA (**D**) mice stained by immunofluorescence with anti-Iba1 antibody. The boxed areas are shown at higher magnification alongside the corresponding brain midsagittal sections. (**E**) Quantification of Iba1 antigen staining by IHC in the brain. The results were expressed as mean ± SEM from two independent biological experiments (*n* = 6 per group). Asterisks represent statistical significance calculated using an unpaired Student’s *t*-test and Welch correction; *P* < 0.05 was considered significant. **P < 0.01.

Microglia/macrophage activation was immunohistochemically assessed in serial brain sections with a pan-macrophage marker, anti-Iba1 (Ionized binding adaptor protein-1), confirming Iba1^+^ microglia/macrophage in the brain parenchyma of both MHV-A59 and MHV-A59+4-PBA mice (Fig. 6A and B). The quantification of staining intensity revealed a significant increase in the activation of Iba1^+^microglia/macrophages in MHV-A59+4-PBA mice compared with MHV-A59-infected mice (Fig. 6E). Immunofluorescence analysis with anti-Iba1 further confirmed increased Iba1 staining throughout the brain of MHV-A59+4-PBA mice when compared with MHV-A59-infected mice that did not receive 4-PBA treatment (Fig. 6C and D).

**Fig 7:**
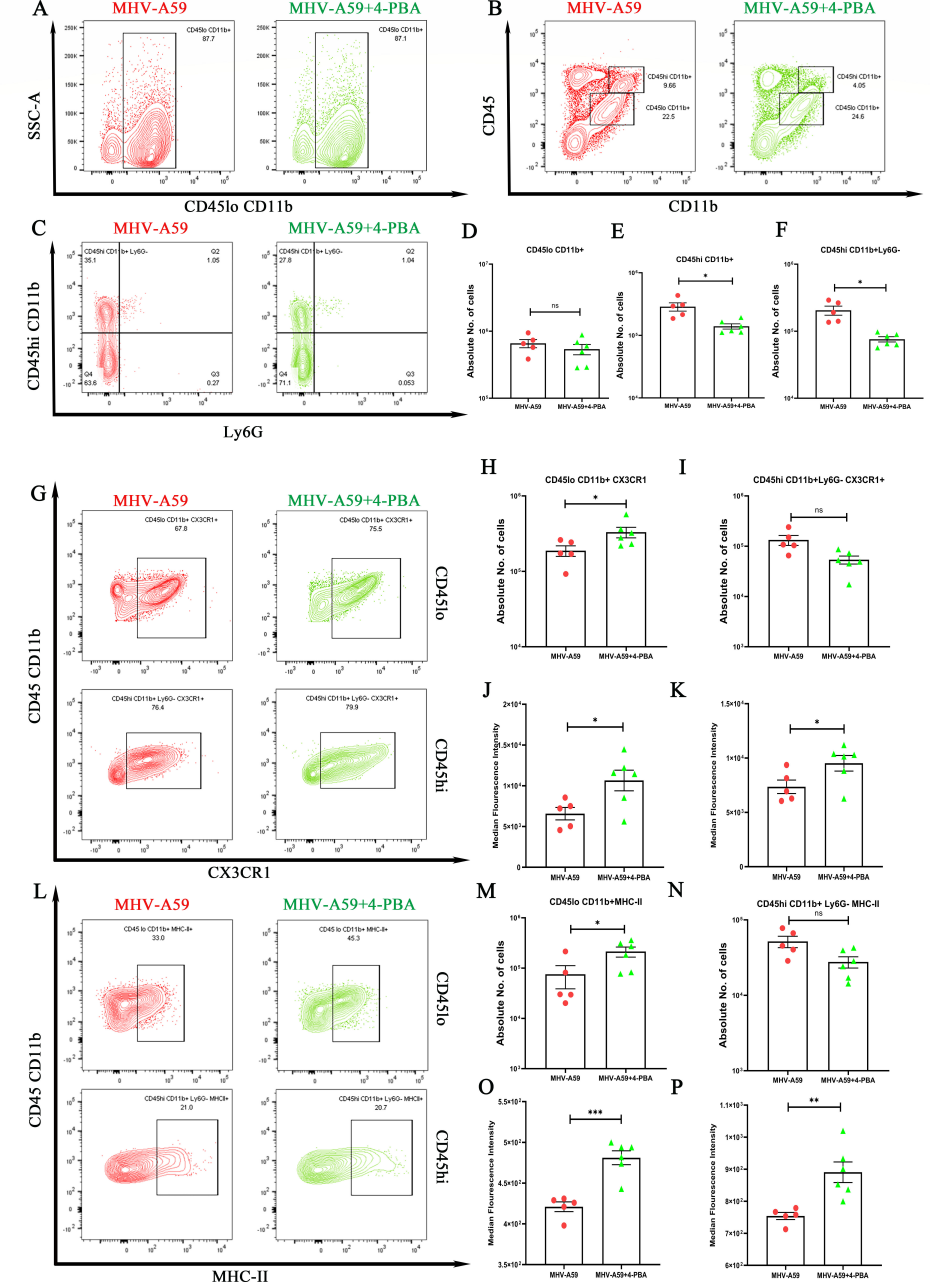
Enhanced activation of peripheral monocyte/macrophage and the brain resident microglia at the acute stage of MHV-A59 infection upon 4-PBA treatment. On day 5 p.i., brains from MHV-A59-infected and MHV-A59+4-PBA-treated mice were harvested for flow cytometry analysis and stained for CD45, CD11b, Ly6G, MHC-II, and CX3CR1. (**A**) Representative flow cytometry plots showing percentages of overall CD45^lo^ CD11b^+^ (brain resident microglia) cell population after gating on live cells, followed by singlets. (**B**) Representative flow cytometry plots showing percentages of overall CD45^lo^ CD11b ^+^ and CD45^hi^ CD11b ^+^ cell population after gating on live cells, followed by singlets. (**C**) Percentages of CD45 and Ly6G-gated cells assessed for CD45^hi^ CD11b^+^Ly6G^-^ (peripheral-derived monocyte/macrophage) presented in flow cytometry plots, (**D**) Absolute cell numbers of CD45^lo^ CD11b^+^ from MHV-A59 and MHV-A59+4-PBA-treated mice. (**E**) Absolute cell numbers of CD45^hi^ CD11b ^+^ from MHV-A59 and MHV-A59+4-PBA-treated mice and (**F**) absolute numbers of CD45^hi^ CD11b^+^ Ly6G^-^ cells represented graphically comparing MHV-A59 and MHV-A59+4-PBA-treated mice. (**G and L**) Representative flow cytometry plots indicating percentages of CD45^lo^CD11b^+^ cells and CD45^hi^ CD11b^+^Ly6G^-^ cells expressing (**G**) CX3CR1 and (**L**) MHC-II comparing MHV-A59 and MHV-A59+4-PBA-treated mice. Graphical representation of absolute numbers of (**H**) CD45^lo^CD11b^+^ CX3CR1^+^ cells and (**I**) CD45^hi^ CD11b^+^ Ly6G^-^ CX3CR1^+^ cells. Scatter plots showing median fluorescence intensity of CX3CR1 expression on (**J**) CD45^lo^ CD11b^+^ CX3CR1^+^ cells and (**K**) CD45^hi^ CD11b^+^Ly6G^-^ CX3CR1^+^ cells comparing MHV-A59 and MHV-A59+4-PBA-treated mice. Graphical representation of absolute numbers of (**M**) CD45^lo^CD11b^+^ MHC-II^+^ cells and (**O**) CD45^hi^ CD11b^+^ Ly6G^-^ MHC-II^+^ cells. Scatter plots showing median fluorescence intensity of MHC-II expression on (**O**) CD45^lo^ CD11b^+^ MHC-II^+^ cells and (**P**) CD45^hi^ CD11b^+^ Ly6G^-^ MHC-II^+^ cells comparing MHV-A59 and MHV-A59+4-PBA-treated mice. The results were expressed as mean ± SEM from two independent biological experiments (*n* = 5–6 per group). Asterisks represent statistical significance calculated using unpaired Student’s *t*-test and Welch correction; *P* < 0.05 was considered significant. *P < 0.05, **P < 0.01, ***P < 0.001, ns-non significant.

We further performed flow cytometric analysis of the whole brain at day 5 p.i. to quantify the absolute numbers of peripheral monocytes/macrophages and brain-resident microglia. Gating on CD45^hi^ and CD45^lo^ allowed the distinction between peripheral infiltrating leukocytes and brain resident immune cells, respectively. The analysis revealed a significant increase in the number of peripheral monocyte/macrophages (CD45^hi^ CD11b ^+^ Ly6G^-^) in MHV-A59-infected mice compared with the MHV-A59+4-PBA-treated mice (Fig. 7B, C, E, and F), whereas the number of brain resident microglia (CD45^lo^ CD11b^+^) (Fig. 7A and D) was comparable between the two groups. Furthermore, we analyzed the expression of MHC-II (major histocompatibility complex class II) and CX3CR1 (C-X3-C chemokine receptor) in these cells. The number of CX3CR1 and MHC II-expressing microglia was significantly higher in MHV-A59+4-PBA-treated groups compared with MHV-A59-infected mice (Fig. 7G, H, L, and M). Furthermore, the median fluorescence intensity (MFI) for CX3CR1 and MHC-II in the microglial population was also significantly higher in the MHV-A59+4-PBA-treated group compared with the MHV-A59-infected group (Fig. 7J and O). Although the absolute number of monocytes/macrophages expressing CX3CR1 and MHC-II did not change significantly between groups, the MFI for CX3CR1 and MHC-II was significantly higher in the MHV A59+4-PBA-treated group compared with the MHV-A59-infected group (Fig. 7G, I, K, L, N, and P). Together, these results suggest enhanced activation of microglia/macrophages in the MHV-A59+4-PBA-treated group compared with the MHV-A59 group.

**Fig 8:**
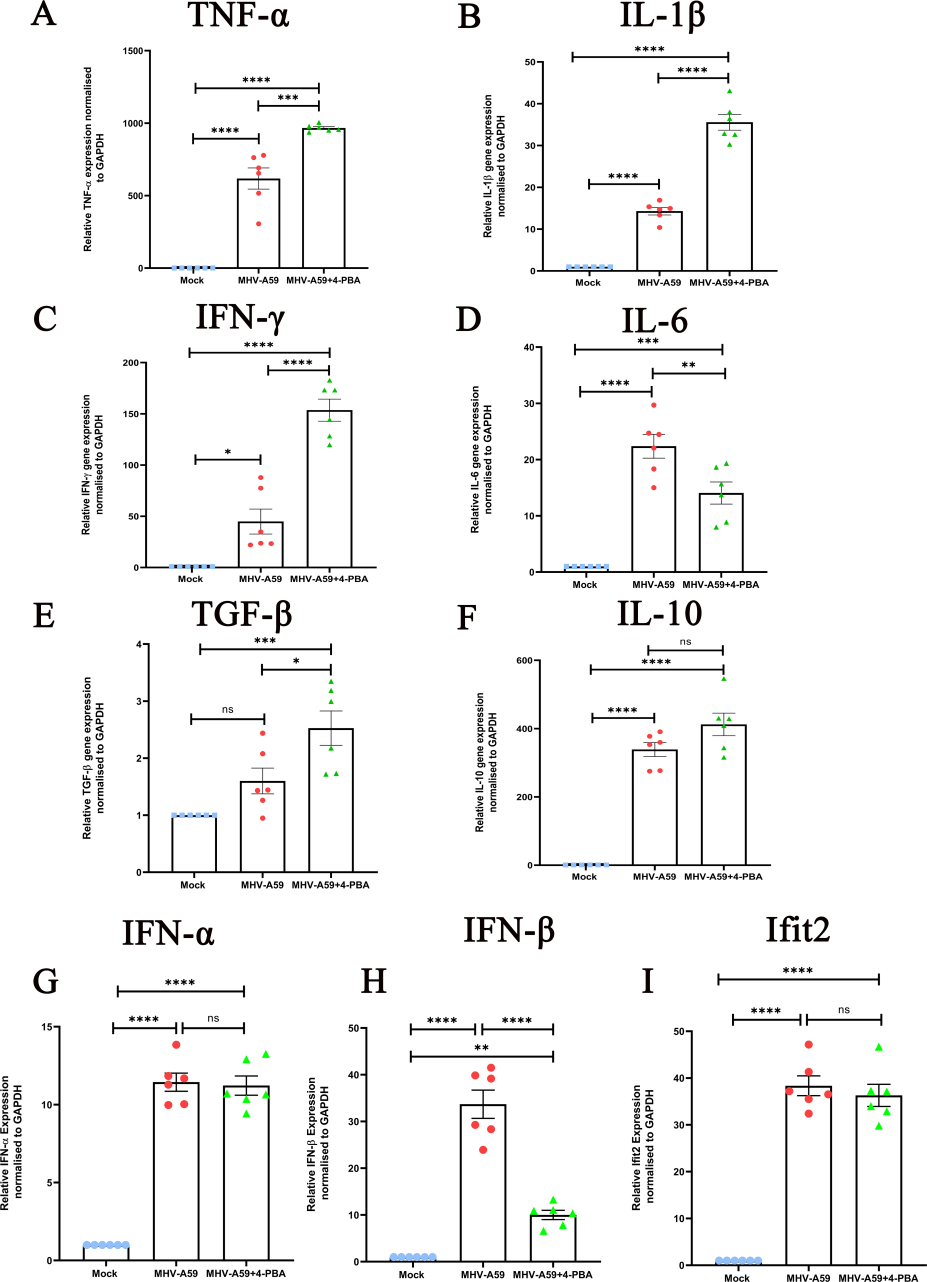
(**A–I**) Differential mRNA expression levels of selected inflammatory cytokines and Ifit2 in brains of infected and 4-PBA-treated mice during the acute stage of infection. RNA extracted from individual brain tissues of MHV-A59-infected and MHV-A59+4-PBA-treated mice at day 5 p.i. was analyzed for mRNA levels of the indicated cytokines and Ifit2 by quantitative PCR. The results were expressed as mean ± SEM (*n* = 6 per group). Asterisks represent statistical significance calculated using ordinary one-way ANOVA. *P* < 0.05 was considered significant. *P < 0.05, **P < 0.01, ***P < 0.001, ****P < 0.0001.

Given this, we studied the inflammatory milieu in the CNS at the acute stage of infection when neuroinflammation is at its peak. Analyzing mRNA levels of selected inflammatory cytokines, we observed that in line with heightened microglia/macrophage activation, mRNA expression levels of TNF-α, IL-1β, IFN-γ, and TGF-β were significantly upregulated in infected brains upon 4-PBA treatment. By contrast, IL-6 and IFN-β were downregulated in MHV-A59+4-PBA mouse brains when compared with those of MHV-A59-infected mice, whereas IFN-α, IL-10, and interferon-induced proteins with tetratricopeptide repeats 2, Ifit2, a type 1 interferon-stimulated gene, did not show any significant difference (Fig. 8A through I). These data suggest a productive, anti-viral immunity during the acute phase of MHV-A59 infection that is enhanced by 4-PBA treatment.

### 4-PBA treatment mitigates the downregulation of Cx43 and ERp29 in the brain upon MHV-A59 infection

**Fig 9:**
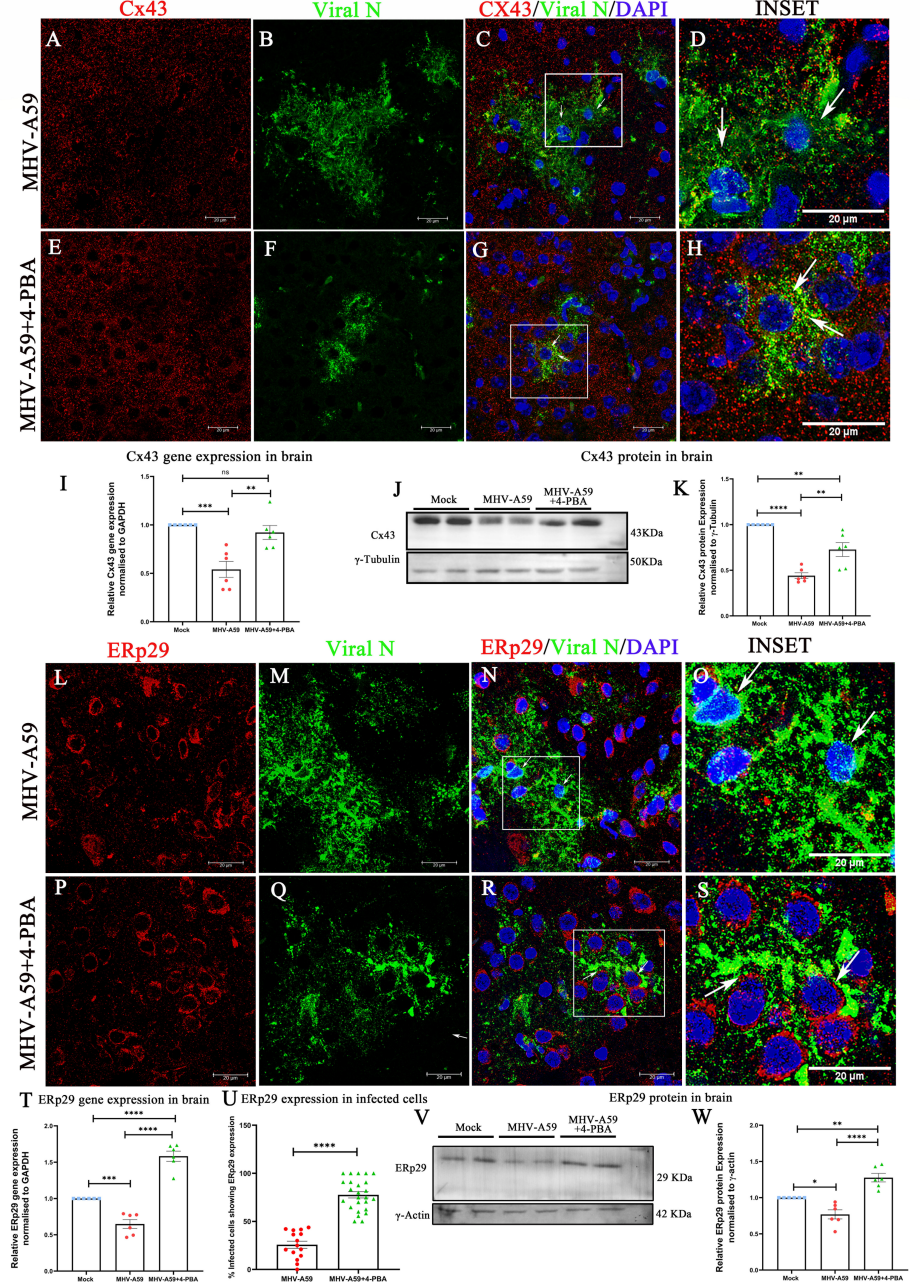
4-PBA treatment maintains Cx43 and ERp29 expression in the brain upon MHV-A59 infection. (A–H) At day 5 p.i expression of Cx43 in the brain of MHV-A59-infected (**A–D**) and MHV-A59+4-PBA-treated (**E–H**) mice was analyzed by double immunolabeling with anti-Cx43 (red) and anti-viral nucleocapsid (viral N) antibodies (green), followed by confocal microscopy. (**C, D and G, H**) Arrows indicate Cx43 labeling in infected cells. (**I**) Scatter plot of differential Cx43 mRNA in MHV-A59–infected and MHV-A59+4-PBA mouse brain measured by qPCR. (**J and K**) Representative immunoblot and corresponding scatter plot of Cx43 protein expression of MHV-A59-infected and MHV-A59+4-PBA-treated mouse brains. (**L–S**) Expression of ERp29 in the brain of MHV-A59-infected (**L–O**) and MHV-A59+4-PBA-treated (**P–S**) mice analyzed by double immunolabeling with anti-ERp29 (red) and anti- viral nucleocapsid (**N**) antibodies (green) followed by confocal immunofluorescence microscopy. (**N, O, and R, S**) Arrows indicate ERp29 labeling in infected cells. (**T**) Scatter plot of differential ERp29 mRNA in Mock, MHV-A59-infected, and MHV-A59+4-PBA mouse brains measured by qPCR. (**U**) Scatter plot for the quantification of the percentage of infected cells expressing ERp29 in infected brain tissue sections from MHV-A59 and MHV-A59+4-PBA treated groups. (**V and W**) representative immunoblot and corresponding scatter plot of Cx43 protein in Mock, MHV-A59-infected, and MHV-A59+4-PBA-treated mouse brains. The results were expressed as mean ± SEM from two independent biological experiments (*n* = 6 per group). Asterisks represent statistical significance calculated using unpaired Student’s *t*-test and Welch correction (when two groups were compared) and ordinary one-way ANOVA when more than two groups were compared); *P* < 0.05 was considered significant. **P < 0.01, ***P < 0.001,****P < 0.0001.

We have previously demonstrated *in vitro* that anti-viral properties of 4-PBA are associated with increased ERp29 expression and improved Cx43 trafficking to the cell surface (7, 16, 17). Thus, we studied the effects of 4-PBA treatment on Cx43 and ERp29 expression in the infected brain *in vivo*. Immunofluorescence analysis using anti-Cx43 (Fig. 9A and E; red) and anti-viral nucleocapsid (Fig. 9B and F, green) antibodies revealed that MHV-A59-infected mice showed a loss of Cx43 expression in infected brain cells (Fig. 9A through D, indicated by arrows). By contrast, MHV-A59+4-PBA-treated mice showed punctate Cx43 expression even in infected cells (Fig. 9E through H; indicated by arrows). MHV-A59-infected mice showed a downregulation of Cx43 mRNA expression compared with mock-infected mice, whereas MHV-A59+4-PBA mice showed significantly higher Cx43 mRNA expression than MHV-A59-infected mice (Fig. 9I). Immunoblot analysis confirmed the downregulation of Cx43 protein levels in MHV-A59-infected mice compared with mock-infected mice, which was mitigated by 4-PBA treatment (Fig. 9J and K). Thus, 4-PBA treatment could alleviate the MHV-A59-mediated downregulation of Cx43 in the brain at the acute stage of infection.

We next investigated the status of ERp29 expression in the infected CNS. MHV-A59 infection in the brain showed a loss of ERp29 in infected cells (Fig. 9L through O, indicated by arrows) as determined by immunofluorescence using anti-ERp29 (Fig. 9L and P; red) and anti-viral nucleocapsid antibodies (Fig. 9M and Q; green). By contrast, immunofluorescence analysis of MHV-A59+4-PBA brains (Fig. 9P through S; indicated by arrows) demonstrated that 4-PBA treatment maintained ERp29 expression even in infected cells (Fig. 9U). In contrast to Cx43, ERp29 was mainly intracellular, consistent with localization to the endoplasmic reticulum (7, 16, 17). The ability of 4-PBA to enhance ERp29 expression was further confirmed by qPCR analysis, which showed that ERp29 mRNA was significantly downregulated upon MHV-A59 infection (Fig. 9T). In addition, 4-PBA treatment in MHV-A59+4-PBA mice showed increased ERp29 that was significantly upregulated when compared with both MHV-A59 and mock-infected mice. A similar trend in expression was observed at the protein level (Fig. 9V and W).

Overall, these results demonstrate that 4-PBA treatment maintains Cx43 expression levels *in vivo*, even in MHV-A59-infected cells in the CNS. MHV-A59 infection in the CNS not only causes downregulation of Cx43 expression but also downregulates the molecular chaperone ERp29. 4-PBA treatment preserves the expression of ERp29 and Cx43, even in MHV-A59-infected cells in the CNS.

### 4-PBA treatment also stabilizes the expression of Cx47 in the infected CNS

**Fig 10:**
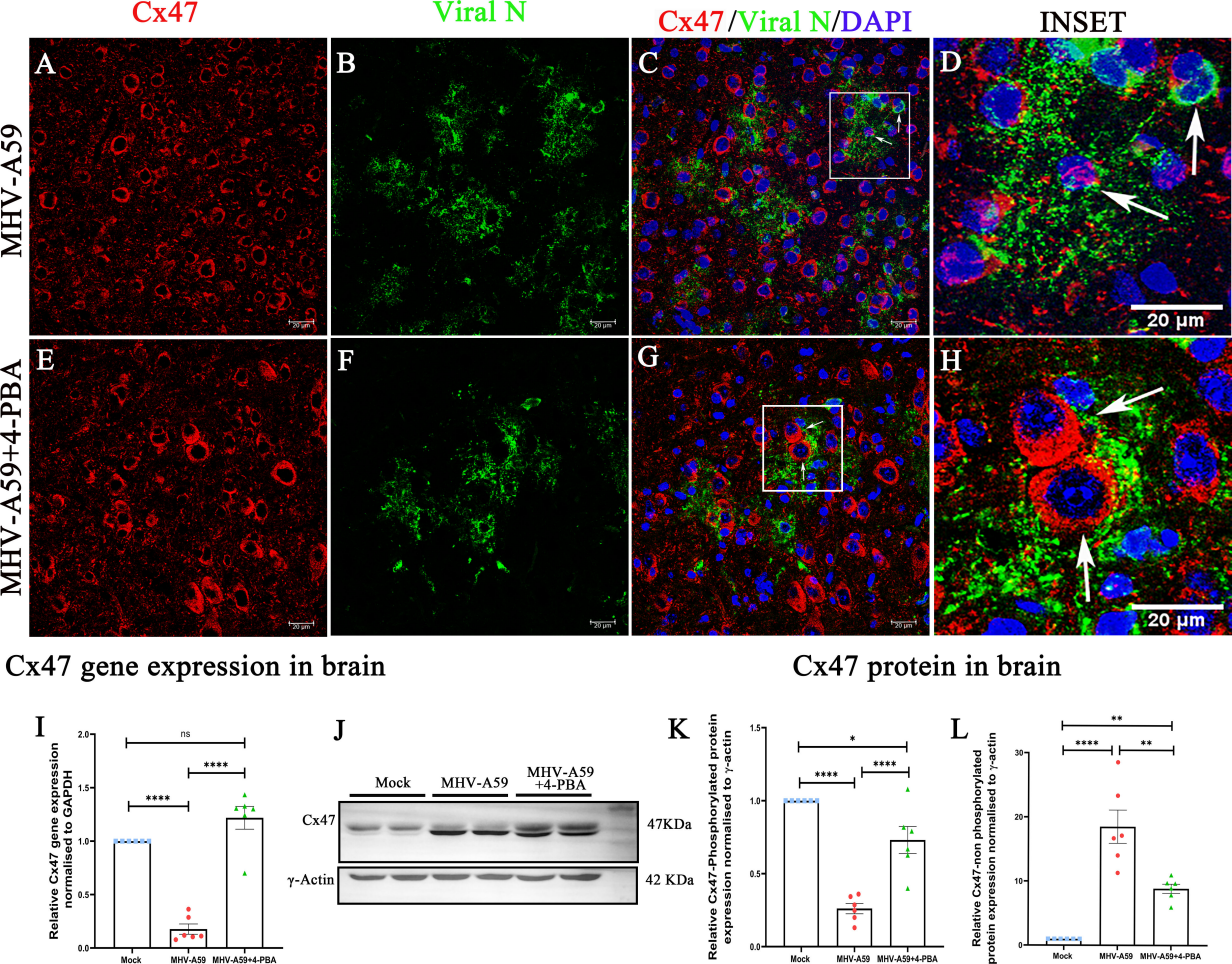
4-PBA treatment stabilizes Cx47 expression in the brain upon MHV-A59 infection. (A–H) Expression of Cx47 in the brain of MHV-A59-infected (**A–D**) and MHV-A59+4-PBA-treated (**E–H**) mice was analyzed by double immunolabeling with anti-Cx47 (red) and anti-nucleocapsid (**N**) antibodies (green), followed by confocal microscopy. (**C, D and G, H**). Arrows indicate Cx47 labeling in infected cells. (**I**) Scatter plot of differential Cx47 mRNA expression in Mock, MHV-A59–infected, and MHV-A59+4-PBA mouse brains measured by quantitative PCR. (**J–L**) Representative immunoblot and corresponding scatter plot of (**K**) Upper band (phosphorylated) and (**L**) lower band (non-phosphorylated) Cx47 protein of MHV-A59-infected and MHV-A59+4-PBA-treated mouse brains. The results were expressed as mean ± SEM from two independent biological experiments (*n* = 6 per group). Asterisks represent statistical significance calculated using ordinary one-way ANOVA. *P* < 0.05 was considered significant. ns - not significant, 0A*P < 0.05, **P < 0.01, ****P < 0.0001.

Additionally, we analyzed the expression of the oligodendrocyte coupling partner of Cx43, Cx47. We analyzed Cx47 expression in the brains of MHV-A59 and MHV-A59+4-PBA mice by immunofluorescence microscopy, using anti-Cx47 (Fig. 10A and E; red) and anti-viral nucleocapsid protein antibodies (Fig. 10B and F; green). Consistent with previous reports (5), Cx47 plaques were disrupted in infected cells in the brain of MHV-A59 infected mice (Fig. 10A through D; indicated by arrows). 4-PBA treatment also rescued Cx47 expression in MHV-A59+4-PBA mice brains at the acute stage of infection (Fig. 10E through H; indicated by arrows). Further analysis of differential gene expression of Cx47 in MHV-A59 and MHV-A59+4-PBA mice revealed that Cx47 gene expression is significantly downregulated upon MHV-A59 infection and that Cx47 downregulation was rescued by 4-PBA treatment. Cx47 gene expression of MHV-A59+4-PBA-infected mice was comparable with mock-infected mice (Fig. 10I).

By immunoblot, Cx47 resolved as a closely migrating doublet of bands, which were quantified separately (Fig. 10J through L), since they may reflect differential Cx47 phosphorylation (22, 23). The upper Cx47 band decreased upon infection with MHV-A59, whereas it did not change much in MHV-A59+4-PBA mice (Fig. 10J and K). By contrast, the lower band increased in MHV-A59-infected mice as well as MHV-A59+4-PBA mice when compared with mock-infected mice, where upregulation was significantly higher in MHV-A59 mice compared with MHV-A59+4-PBA mice (Fig. 10J and L). Thus, 4-PBA treatment enhanced Cx47 expression in addition to Cx43 and ERp29.

### 4-PBA treatment protects mice from chronic virus-induced inflammatory demyelination

We then determined whether 4-PBA treatment could also influence the chronic stage of demyelination in MHV-A59 infection. To study this, we treated mice with 200 mg/kg body weight/day 4-PBA until day 10 p.i. with parallel MHV-A59-infected mice that did not receive any treatment, as described earlier (Fig. 2B). The mice were sacrificed at day 30 p.i., and tissues were harvested to investigate demyelination at this stage.

**Fig 11:**
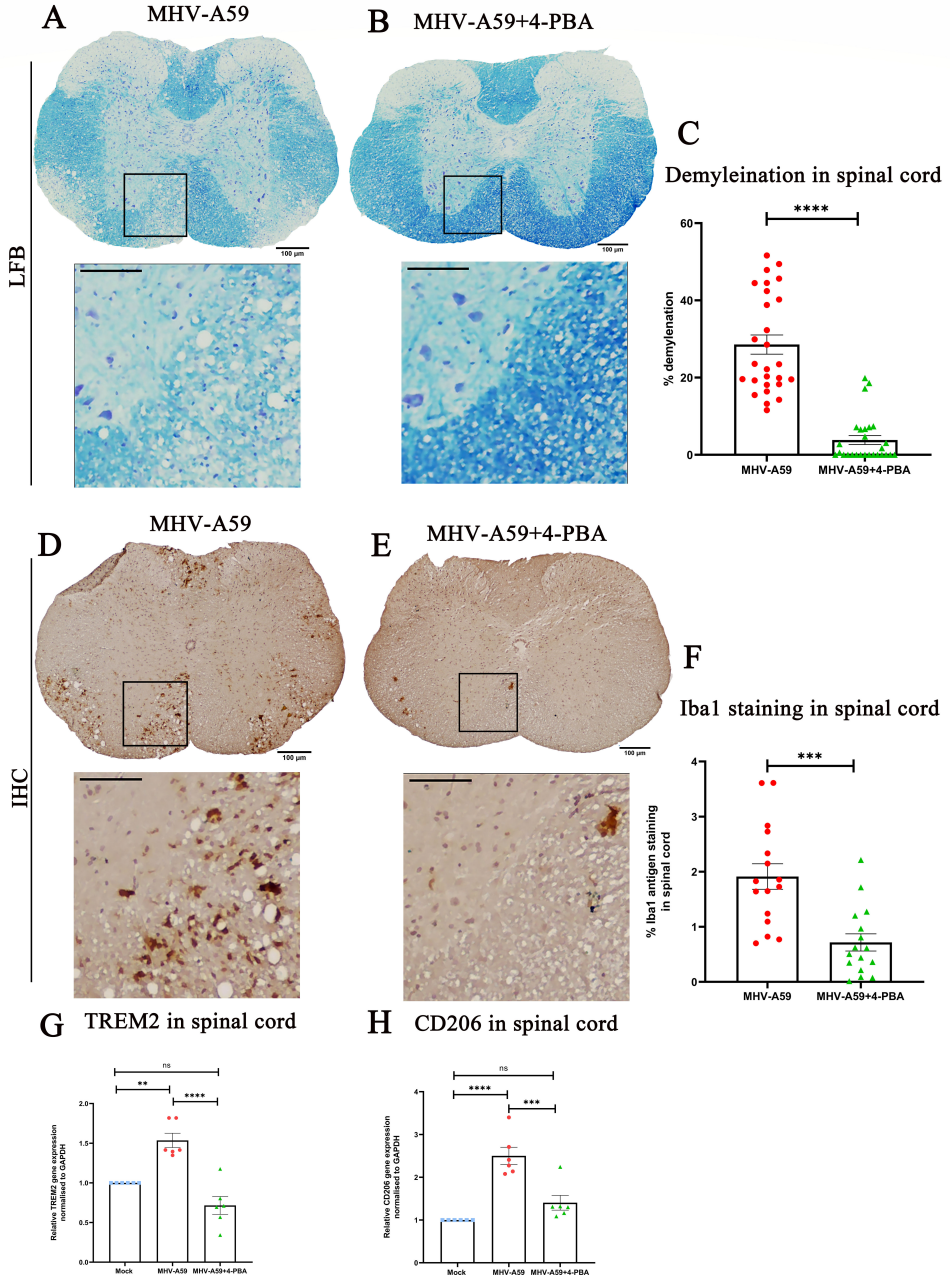
4-PBA treatment mitigates MHV-A59-mediated chronic stage demyelination. (**A**) On day 30 p.i., cross-sections of MHV-A59-infected (**A and D**) and MHV-A59+4-PBA-treated (**B and E**) mouse spinal cords were analyzed for demyelination by LFB (A, B) and the presence of microglia/macrophages in the demyelinating lesions for Iba1 by immunohistochemistry (**D and E**). Black-boxed areas represent higher magnification below the corresponding spinal cord cross-sections. Scale bar 100 µm, 50 µm. Quantification of (**C**) percent area of demyelination and (**F**) Iba1 staining intensity. The relative transcript abundance of (**G**) TREM-2 and (**H**) CD206 was analyzed in the infected spinal cords using qPCR and compared between mock, MHV-A59-infected, and MHV-A59+4-PBA treated on day 30 p.i. The results were expressed as mean ± SEM from two independent biological experiments (*n* = 6 per group). Asterisks represent statistical significance calculated using unpaired Student’s *t*-test and Welch correction (when two groups were compared) and ordinary one-way ANOVA when more than two groups were compared); *P* < 0.05 was considered significant. ns-not significant, *P < 0.05, ***P < 0.001, ****P < 0.0001, ****P < 0.0001.

Luxol Fast Blue (LFB) staining performed on spinal cord sections of MHV-A59 and MHV-A59+4-PBA-treated mice revealed significantly lower demyelination in 4-PBA-treated mice (Fig. 11B) compared with MHV-A59-infected mice (Fig. 11A). The demyelination quantification revealed that the percentage of demyelination in MHV-A59+4-PBA-treated mice was significantly lower compared with MHV-A59-infected mice (Fig. 11C). Since microglia/macrophage-mediated myelin stripping is the primary mechanism of demyelination in the MHV-induced chronic demyelination model, we next investigated the microglia/macrophage activation in spinal cords of these mice (24). The demyelinating plaques showed a distinct accumulation of Iba1^+^ microglia/macrophages in serial spinal cord sections of MHV-A59-infected mice (Fig. 11D), whereas the Iba1 staining was lower in the spinal cords of MHV-A59 +4-PBA-treated mice (Fig. 11E). Quantification of the Iba1 antigen staining by immunohistochemistry shows significantly reduced Iba1 staining in the spinal cords of MHV-A59+4-PBA-treated mice compared with MHV-A59-infected mice (Fig. 11F). RNA from day 30 p.i. spinal cords was subjected to qPCR analysis to assess microglial phagocytic marker transcript levels of TREM2 and CD206. There was reduced mRNA expression of TREM2 (Fig. 11G) and CD206 (Fig. 11H) in spinal cords of MHV-A59+4-PBA-treated mice compared with MHV-A59-infected mice. Thus, these results demonstrate that 4-PBA treatment effectively protects mice against MHV-A59-mediated chronic stage demyelination.

### Inhibition of Cx43 compromises the antiviral potential of 4-PBA

To determine whether the reduced viral susceptibility observed following 4-PBA treatment was mediated through Cx43, we took a reductionist approach and inhibited Cx43 *in vitro* in primary astrocytes. To inhibit Cx43, we employed a Cx43 mimetic peptide, Gap 27, a well-established inhibitor of Cx43 gap junctional communication (25–27).

**Fig 12:**
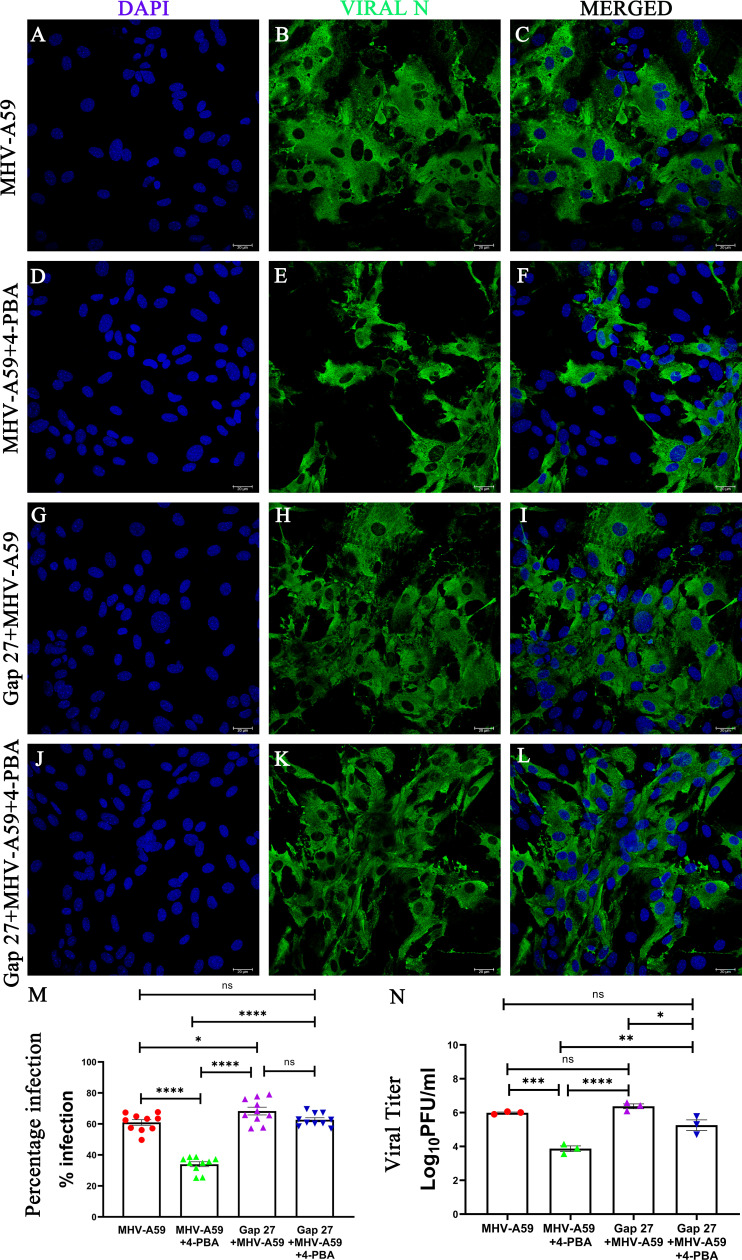
4-PBA loses antiviral potency upon Cx43 inhibition. Representative confocal photomicrographs of murine primary astrocytes infected with MHV-A59 for 24 h at MOI 5 and analyzed by immunolabeling with anti-nucleocapsid (Viral-N) antibody (green) and mounted in a DAPI (blue)-containing mounting medium following (**A–C**) MHV-A59 infection, (**D–F**) MHV-A59 infection+4-PBA treatment, (**G–I**) Gap 27 treatment+MHV-A59 infection, and (**J–L**) Gap 27 treatment+MHV-59 infection and 4-PBA treatment (Scale Bar = 20 µm). (**M**) Scatter plots of the quantification of percentage infected nuclei, respectively, calculated from 10 fields from three independent experiments. (**N**) Viral titer analyzed from supernatant of MHV-A59-infected, MHV-A59+4-PBA treated, Gap 27+ MHV-A59-infected, and Gap 27+ MHV-A59+4-PBA-treated primary astrocytes. The results were expressed as mean ± SEM (*n* = 3 per group). Asterisks represent statistical significance calculated using ordinary one-way ANOVA; *P* < 0.05 was considered significant (*P < 0.05, **P < 0.01, ***P < 0.001, ****P < 0.0001, ns-not significant).

As demonstrated earlier, 4-PBA treatment (MHV-A59+4-PBA) significantly reduced MHV-A59 infectivity in murine primary astrocytes compared with MHV-A59-infected cells that did not receive 4-PBA treatment (MHV-A59) (Fig. 12D through F and A through C, M). Although primary murine astrocytes pre-treated with Gap 27 (Gap 27+MHV-A59) exhibited an increase in the MHV-A59 infectivity compared with its untreated control (MHV-A59) (Fig. 12G through I and A through C, M), indicating a loss of the protective effect observed with intact Cx43-mediated coupling.

Furthermore, when astrocytes were pre-treated with Gap 27, infected with MHV-A59, and treated with 4-PBA, the anti-viral effect of 4-PBA was notably diminished (Fig. 12J through L, M), confirming that Cx43 is a key mediator of the reduced viral susceptibility conferred by 4-PBA. These findings strongly suggest that the integrity of Cx43 gap junctional communication is crucial for 4-PBA-mediated antiviral effect during MHV-A59 infection. Furthermore, the viral titer was determined for the supernatant of these cell groups. 4-PBA treatment (MHV-A59+4-PBA) significantly reduced MHV-A59 infectivity in murine primary astrocytes compared with MHV-A59-infected cells that did not receive 4-PBA treatment (MHV-A59), with a 2-log reduction in infection upon 4-PBA treatment (Fig. 12N). Furthermore, when astrocytes were pre-treated with Gap 27, infected with MHV-A59, and treated with 4-PBA, the anti-viral effect of 4-PBA was notably diminished, with only a 1-log reduction in infection (Fig. 12N). Thus, the viral titer data are consistent with that of the presented immunofluorescence analysis, suggesting that inhibition of Cx43 diminishes 4-PBA’s protective effect.

These *in vitro* findings are consistent with our *in vivo* observations, where 4-PBA treatment enhanced Cx43 levels and was associated with the restricted viral spread and attenuated CNS pathology in MHV-A59-infected mice.

## DISCUSSION

This study uncovers four pivotal and previously unreported findings: (i) 4-PBA effectively restricts the *in vitro* and *in vivo* spread and infectivity of MHV-A59 at the acute stage Day 5 p.i., (ii) acute-MHV-A59 infection in the CNS downregulates the expression of ERp29, a Cx43 molecular chaperone, (iii) 4-PBA treatment was able to maintain expression of ERp29, Cx43, and Cx47 even in infected cells at an acute stage in the MHV-A59-infected CNS, and (iv) 4-PBA significantly reduces the MHV-A59 infection-mediated virus-induced neuroinflammatory demyelination at the chronic stage of infection.

Our study is the first to demonstrate the antiviral efficacy of 4-PBA against β-coronavirus infection *in vivo* in the mouse CNS. The novelty of this study lies in demonstrating the *in vivo* anti-viral potential of 4-PBA against murine β-coronavirus MHV-A59 is likely through a mechanism involving ERp29 and Cx43. 4-PBA has demonstrated anti-viral effects against the Japanese Encephalitis Virus, Hepatitis C virus, and Herpes simplex type 1 virus through mechanisms such as ER stress inhibition, histone deacetylase inhibition, and CREB3 downregulation, respectively (28–30). The ability of 4-PBA to stabilize the expression of ERp29, and thus, gap junction proteins Cx43 and Cx47 *in vivo* in the mouse CNS, represents a unique and previously unreported finding.

In a previous *in vitro* study, we demonstrated that 4-PBA rescues Cx43 expression and trafficking, which restricted MHV-A59 infectivity (7). Following this, a recent study in La Crosse virus infection also harnessed 4-PBA, demonstrating induction of Cx43 expression and reduced viral load at early stages of infection; however, the mechanism driving 4-PBA-mediated induction of Cx43 expression remains unexplored (31). Our study addresses this gap by demonstrating that 4-PBA-mediated improvement in Cx43 expression correlated with enhanced ERp29 expression, a well-known molecular chaperone of Cx43. Previously using DBT cells, we demonstrated that DBT cells overexpressing ERp29 were resistant to the deleterious effects of MHV-A59 infection owing to improved Cx43 trafficking and expression in these cells, consistent with ERp29-mediated Cx43 expression being a mechanism of action for the effects of 4-PBA *in vivo* (7).

The previously available literature has clearly demonstrated that 4-PBA stimulates ERp29 expression that primarily acts at the level of cellular trafficking of Cx43 to the cell surface rather than enhancing its transcription (7, 17, 20). Our previous *in vitro* studies have demonstrated that in infected conditions, when Cx43 is unable to traffic properly, misfolded protein accumulates, which can trigger a feedback mechanism leading to overall retention and transcriptional downregulation of Cx43 (7, 17, 21). When 4-PBA, via ERp29, facilitates proper trafficking of Cx43 to the cell surface, this restores normal cellular feedback, resulting in normalization of Cx43 gene expression. Similarly, in the current study, we observe that Cx43 expression is only restored to its basal levels and does not increase beyond that. The partial restoration of Cx43 mRNA levels likely reflects the relief of infection-induced transcriptional repression of Cx43. This effect represents normalization to homeostatic levels rather than active transcriptional upregulation by 4-PBA.

Upon intracranial infection with 2000 PFU of MHV-A59 (1/2 LD50) in 4-week-old old-C57BL/6 mice, 24 h post-infection, MHV-A59 virus particles are found in the meninges, ependyma, and choroid plexus. This is followed by viremia on the second and third days p.i., consequently spreading from the brain to the liver, causing hepatitis (32). Thus, the observed hepato-protective effects of 4-PBA in our study are likely a secondary consequence of decreased CNS viral burden, resulting in fewer viral particles entering the circulation via viremia, thereby reducing viral seeding of the liver and attenuating hepatic pathology. Future studies will be designed to involve direct intrahepatic MHV-A59 inoculation to understand if 4-PBA exerts its protective effects beyond the CNS.

The dissociation between microglia and astrocytes activation reveals an interesting contrast in neuroinflammatory dynamics. Specifically, we observed reduced astrocyte activation in MHV-A59+4-PBA-treated mice compared with MHV-A59-infected mice, which aligns with the lower viral load detected in these mice and thus reflects the reduced astrogliosis. In contrast, microglial activation was heightened in the MHV-A59+4-PBA-treated group, suggesting a temporal divergence in the activation profile of these glial populations. Microglia typically have a lower threshold for activation than astrocytes and are known to respond rapidly and robustly to even minor pathological challenges or tissue alterations. Moreover, their activation tends to be more sustained over time (33). Our current study focused on day 5 post-infection, the time point at which viral load peaks in this MHV-A59 model, to assess the antiviral efficacy of 4-PBA. However, future studies should explore earlier time points to determine whether astrocyte activation is initially heightened in the MHV-A59+4-PBA group. This would help clarify whether the reduced astrocytic response observed at day 5 reflects an early transient activation phase followed by resolution.

In models of hypoxia/reoxygenation injury, spinal cord injury, and Alzheimer’s disease, the deletion of Cx43 has been associated with reduced microglial activation (34–36). Furthermore, in the model of spared nerve injury-induced neuropathic pain, astrocyte Cx43 played a significant role in regulating microglial activation (37). This suggests that increased Cx43 levels upon 4-PBA treatment likely enhance microglial activation, leading to increased secretion of inflammatory cytokines, mounting a robust inflammatory response that aids viral clearance.

Previous studies in MHV have demonstrated that reduced immune cell infiltration in the CNS of Ifit2^−/−^ and CD4^−/−^ mice led to aggravated chronic stage demyelination (38–40). In MHV-infected CD40L^−/−^ mice, the reduced interaction between CD4^+^ T cells and microglia resulted in reduced microglial activation and CD4^+^ T cell infiltration in the CNS, causing failure of glial cells to restore homeostasis, leading to persistently activated microglia and macrophages, thereby worsening chronic demyelination pathology (41). Thus, highlighting the need for an effective transition from innate to adaptive immunity and ensuring the restoration of CNS homeostasis. Cx43 is important for efficient adaptive immune response in the CNS, facilitating antigen cross-presentation at the immunological synapse, critical for adaptive immune responses and effective T-cell activation (42). The observed enhanced Iba1 activation at day 5 p.i. and reduced chronic-stage demyelination at day 30 p.i. in 4-PBA-treated mice suggests an effective resolution of inflammation following acute-stage viral clearance. Detailed studies on immune cell infiltration across various stages of infection can further elucidate how 4-PBA-mediated modulation of Cx43 influences adaptive immune response and viral clearance. These findings underscore the complexity of defining Cx43’s role in neuroinflammation and its potential as a therapeutic target, balancing the need for inflammation to clear pathogens with the necessity of resolving inflammation to prevent chronic CNS damage. Another mechanism by which 4-PBA can reduce MHV-A59-induced chronic-stage demyelination and axonal loss is by stabilizing the expression of the oligodendrocyte Cx47, improving Cx43-Cx47-mediated GJIC, which is important for proper myelination in humans as well as rodents (4). In patients with Balo’s disease, a concentric variant of MS, astrocytic Cx43 and oligodendrocyte Cx43/Cx47 expression was diminished in both demyelinated and preserved myelin layers. NMO and MS patient samples showed preferential loss of astrocytic Cx43 in actively demyelinating and chronic active lesions, where heterotypic Cx43/Cx47 astrocyte-oligodendrocyte gap junctions were lost (43–45). Such changes were also observed in important animal models of MS, EAE, and MHV.

In myelin oligodendrocyte glycoprotein (MOG) and myelin basic protein (MBP)-induced experimental autoimmune encephalomyelitis (EAE), levels of astrocytic Cx43 and Cx47 on mature oligodendrocytes were significantly reduced at an early stage of EAE, whereas Cx43 expression was increased at late EAE stages (46). In this model, treatment with a pharmacological blocker of Cx43 hemichannels in mice, INI-0602, attenuated acute and chronic EAE (47). However, it should be considered with caution that these models typically lack a pathogen or infectious agent, where inflammation is crucial for pathogen clearance. Thus, reducing inflammation in these non-infectious models might be therapeutic. However, in infectious models of demyelination such as MHV-A59, initial acute inflammation is necessary for viral clearance. The inflammation must, however, resolve once the virus is cleared, as unresolved inflammation in the CNS can have devastating consequences, including demyelination and impaired remyelination.

Thus, overall, the study highlights 4-PBA as an important therapeutic agent for restricting murine β-coronavirus spread and infectivity in mouse CNS as well as for reducing viral triggers and associated demyelination for MS.

## MATERIALS AND METHODS

### Isolation of primary mixed glial culture and enrichment of astrocytes

Primary mixed glial cell cultures were established from postnatal day 0 to 1 mouse pups, with minor modifications as previously described (48, 49). Briefly, following the removal of meningeal covering, brain tissues were homogenized and subjected to enzymatic digestion by incubating in a rocking water bath at 37°C for 30 min in HBSS containing 300 µg/mL DNase I and 10 mg/mL trypsin. Dissociated cells were triturated with 0.25% of FBS in HBSS and passed through a 70 µm nylon mesh. Cells were washed in HBSS at 300 g for 10 min and plated in astrocyte growth medium containing DMEM supplemented with 10% FBS, 1% L-glutamine, and 1% penicillin and streptomycin. After 24 h, the media were changed to remove all nonadherent cells, and the cells were allowed to grow to confluency with media changed every 3 days thereafter. After 9–10 days, once the cultures had become confluent, the addition of fresh growth medium was stopped for 10 days to allow differential adhesion of astrocytes and microglia. Following this, the culture flasks were thoroughly agitated in an orbital incubator shaker (200 rpm for 40 min at 37°C), followed by a quick shaking to remove the loosely adherent microglial cells by using the differential adherent properties of astrocytes and microglia. The remaining adherent monolayers were enriched in astrocytes.

### Infection of primary astrocytes with MHV-A59 and 4-PBA treatment

Primary astrocytes were infected with inoculation medium (DMEM containing 1% penicillin-streptomycin and 1% glutamine with 2% FBS) containing MHV-A59 at multiplicities of infection (MOI) of 5 and allowed to adhere for 1.5 h at 37°C in a humidified CO_2_ incubator. After 1.5 h of incubation, the inoculum was removed, and infected cells were maintained in an astrocyte-specific medium containing 10% normal FBS (−4-PBA) or in an astrocyte-specific medium containing 10% FBS supplemented with 10 mM 4-PBA for 24 h. At 24 h p.i., the cell culture medium was removed, and the cells were fixed using 4% paraformaldehyde (PFA).

### Immunofluorescence of primary astrocytes

Immunofluorescence studies were performed according to the protocol described previously (6), with minor modifications. Primary astrocytes were plated on etched glass coverslips and fixed with 4% paraformaldehyde (PFA). Permeabilization was done with phosphate-buffered saline (PBS) containing 0.5% Triton X-100 and blocked with PBS containing 0.5% Triton X-100 and 2.5% heat-inactivated goat serum (PBS-GS). The cells were incubated with primary antisera diluted in blocking solution for 1 h, washed, and then labeled with secondary antisera diluted in blocking solution. Cells were then washed with PBS, mounted with mounting medium containing 4′,6-diamidino-2-phenylindole (DAPI; VectaShield, Vector Laboratories), and visualized using a Zeiss confocal microscope (LSM710). Images were acquired and processed with Zen2010 software (Carl Zeiss).

### Virus, inoculation of mice, and experimental design

MHV-free, 4-week-old, C57BL/6 male mice were intracranially inoculated with one-half of the 50% lethal dose (one-half LD_50_) of MHV-A59 (2,000 PFU). The mice were monitored daily for weight change, signs, and symptoms of disease. Severity of disease pathology was graded regularly using the following scale: 0, no disease symptoms; 0.5, ruffled fur, possibly slower movement; 1.0, hunched back position with mild ataxia, possibly slower movement; 1.5, hunched back position with mild ataxia and hind-limb weakness, restricted movement; 2, ataxia, balance problem and/or partial paralysis but still able to move; 2.5, one leg completely paralyzed, motility issue but still able to move around with difficulties; 3, severe hunching/wasting/both hind limb paralysis and mobility is severely compromised; 3.5, severe distress, complete paralysis, and moribund; and 4, dead (50).

Mice were mock-infected with PBS-BSA and were maintained in parallel. Mice were sacrificed at the peak of inflammation/acute stage (day 5 p.i.) and the peak of demyelination/chronic stage (day 30 p.i.). Brain, spinal cord, and liver tissues were harvested for experimentation.

Mice were divided into two groups: one group (MHV-A59) received intracranial (IC) inoculation with MHV-A59 (50% of the LD_50_ dose: 2,000 PFU) on day 0. The second group (MHV-A59+4- PBA) was infected with the same dose of MHV-A59 and an intraperitoneal (IP) injection of 4-PBA at 200 mg/kg of body weight 0.5 hrs prior to IC inoculation. Additionally, the MHV-A59+4-PBA group received daily 4-PBA injections of 200 mg/kg of body weight/day until day 4 for acute stage studies and until day 10 for chronic stage studies (Fig. 2A and B).

The rationale behind the dosing strategy was derived from previous studies by our group. In the MHV-A59-induced model, we observe that the viral infection in the brain peaks at Day 5 post-infection (1). At this point, we observe downregulation of Cx43 as well. After Day 5 p.i., the infectious viral particle starts decreasing, with most of the infectious viral particles being cleared out by Day 10 p.i. At this point of Day 10 p.i., we observe that Cx43 expression also returns to its normal basal levels (5). Given the role of 4-PBA in rescuing Cx43 levels, we administered 4-PBA daily up to Day 10 p.i., aligning with the period when Cx43 is most affected. Extending 4-PBA treatment beyond Day 10 would likely be redundant. To balance therapeutic benefit with judicious drug use, we limited the treatment to the period of maximal need and discontinued it between days 10 and 30 p.i.

Vehicle control mice (that received IP PBS injection) were also maintained in parallel. Vehicle control-treated infected mice showed no differential phenotypic or histopathological features compared with MHV-A59-infected mice (data not shown).

4-PBA solution was prepared by titrating equimolecular amounts of 4-phenylbutyric acid (Sigma, Madrid, Spain) and sodium hydroxide to pH 7.4. The dose was chosen in reference to a previous dose-response study performed using increasing doses of PBA from 100 to 800  mg/kg to determine the most efficacious dose (51, 52).

### Estimation of viral replication

MHV-A59 and MHV-A59+4-PBA mice were sacrificed at Day 5 p.i. for the estimation of infectious viral particles. Brain and liver tissues were harvested and placed into 1 mL of isotonic saline containing 0.167% gelatin (gel saline). Tissues were weighed and kept frozen at −80°C until titered. Tissues were subsequently homogenized, and using the supernatant, the viral titers were quantified by standard plaque assay protocol on tight monolayers of L2 cells as described previously (53) using the formula: plaque-forming units (PFUs) = (no. of plaques × dilution factor/ml/gram of tissue) and expressed as log_10_ PFUs/gram of tissue.

The viral titer for primary astrocytes (MHV-A59, MHV-A59+4-PBA, Gap 27+ MHV-A59, Gap27+MHV-A59+4-PBA) was determined from the culture supernatants as described above using the formula: plaque-forming units (PFUs) = (no. of plaques × dilution factor/mL) and expressed as Log_10_PFUs/mL.

### Histopathology and immunohistochemical analysis

Liver, brain, and spinal cord tissues were harvested and embedded in paraffin following transcardial perfusion with PBS. Tissues were post-fixed in 4% paraformaldehyde for 36–48 h, following which tissues were processed in increasing concentrations of ethyl alcohol, xylene, and paraffin wax, embedded in paraffin, and sectioned into 5 µm thick transverse sections (liver and spinal cord) and mid-sagittal sections of the brain containing olfactory bulb, cerebral cortex, hippocampus, hypothalamus, midbrain, cerebellum, and part of brain stem using Thermo Scientific HM 355 S sectioning system. Liver sections were stained with hematoxylin and eosin for histopathologic analysis. Moreover, spinal cords were also stained with Luxol fast blue (LFB) to evaluate for demyelination as described previously (24).

Immunohistochemical staining of the brain and spinal cord tissue serial sections was performed as described previously, using the avidin-biotin immunoperoxidase technique (Vector Laboratories) with 3, 3-diaminobenzidine as the substrate (54). The following primary antibodies were used: anti-Iba-1 (Wako), anti-GFAP (Sigma-Aldrich, MO, USA), monoclonal antibody directed against the nucleocapsid protein (N) of MHV (monoclonal antibody clone 1-16-1 provided by Julian Leibowitz, Texas A&M University), anti-Cx43 (Sigma-Aldrich, MO, USA), and anti-ERp29 (Invitrogen, MA, USA) (Table 1). Control slides from mock-infected and vehicle-control mice were stained in parallel. All the slides were coded and read blindly for subsequent analyses.

**TABLE 1:** Primary antibodies used in the study

Sl. no.	Primary antibody	Application	Dilution	Source
1	Anti-Cx43 (polyclonal)	IF WB IHC	1:1,000 1:1,000 1:1,000	Sigma-Aldrich, MO, USA
2	Anti-GFAP (polyclonal)	IF IHC	1:500 1:500	Sigma-Aldrich, MO, USA
3	Anti-Cx47 (polyclonal)	IF WB	1:500 1:1,000	Invitrogen, MA, USA
4	Anti-Iba1 (polyclonal)	IF IHC	1:500 1:500	Wako
5	Anti-viral nucleocapsid (monoclonal)	IF IHC	1:40 1:40	Monoclonal antibody clone 1-16-1 provided by Julian Leibowitz, Texas A&M University
6	Anti-ERp29 (polyclonal)	IF WB IHC	1:500 1:1,000 1:500	Invitrogen, MA, USA

### Quantification of histopathological sections

Image analysis was performed using the basic densitometric thresholding application of Fiji (Image J, NIH Image, Scion Image) as described previously (55) Briefly, image analysis for IHC of Iba1, GFAP, and viral nucleocapsid staining in sections was performed by capturing the images at the highest magnification (4× for brain, 10× for spinal cord) such that the entire section (i.e., scan area) can be visualized within a single frame. The RGB image was deconvoluted into three different colors to separate and subtract the DAB-specific staining from the background hematoxylin staining. The perimeter of each brain and spinal cord tissue was digitally outlined, and the area was calculated in μm^2^. A threshold value was fixed for each image to ensure that all antibody-marked cells were considered. The amount of Iba1, GFAP, and viral nucleocapsid staining was calculated as the “% area of staining.”

To determine the area of demyelination, LFB-stained spinal cord cross-sections from each mouse were chosen and analyzed using Fiji software (Image J, NIH Image, Scion Image) as described previously (55, 56). Briefly, the total perimeter of the white matter regions in each cross-section was marked and calculated by adding together the dorsal, ventral, and anterior white matter areas in each section. Also, the total area of the demyelinated regions was outlined and collated for each section separately. The percentage of spinal cord demyelination per section per mouse was calculated (5–7 sections from the cervical and thoracic regions of the spinal cord/mouse were examined).

### Flow cytometry analysis

Flow cytometry analysis was performed according to the protocol described previously (39, 41) with minor modifications. Mice were transcardially perfused with PBS, and brains were homogenized in 2 mL of RPMI containing 25 mM HEPES (pH 7.2), using Tenbroeck tissue homogenizers. Following centrifugation at 450 *× g* for 10 min, the cell pellets were resuspended in RPMI containing 25 mM HEPES, adjusted to 30% Percoll (Sigma), and underlaid with 1 mL of 70% Percoll. Following centrifugation at 800 × *g* for 30 min at 4°C, the cells were recovered from the 30%–70% interface, washed with RPMI, and suspended in FACS buffer (0.5% bovine serum albumin in Dulbecco’s PBS). Cells were counted using an automated cell counter (Invitrogen) to obtain the number of total leukocytes. One million cells were stained for flow cytometry.

Specific cell types in the brain were identified by staining with fluorochromes like fluorescein isothiocyanate (FITC), phycoerythrin ([PE], [PECy7]), peridinin chlorophyll protein ([PerCP, PerCpCy5.5]), allophycocyanin ([APC, APCCy7]), and violet excitable dyes ([V450, V500]) conjugated MAb for 45 min at 4°C in FACS buffer. Expression of surface markers was characterized with MAb (all from BD Biosciences except where otherwise indicated) specific for CD45 (clone Ly-5), CD11b (clone M1/70), CX3CR1 (clone SA011F11, Biolegend), MHCII (clone 2G9), and Ly-6G (clone 1A8).

Samples were acquired on a BD LSR Fortessa X-20 flow cytometer (BD Biosciences) and analyzed on FlowJo 10 software (Treestar, Inc., Ashland, OR). First, the cells were gated based on forward scatter (FSC) and side scatter (SSC) to focus on live cells, and then doublet exclusion using FSC-A and FSC -W was performed. Furthermore, the cells were analyzed to differentiate myeloid and lymphoid populations. Myeloid cells were gated from a primary gating on CD45, and an additional CD3 gating was applied for the lymphoid populations. Single colors were used in all the experiments. Beads were gated based on their FSC/SSC pattern.

### Protein isolation and immunoblot analysis

On average, 150 mg of whole brain tissue was collected from euthanized mice following transcardial perfusion with 20 mL PBS and flash-frozen in liquid nitrogen. Tissue was then lysed in 1 mL of RIPA buffer (0.1% SDS and 0.1% Triton X-100) with 1× complete mini protease inhibitor cocktail tablets (#11836153001; Roche) and phosphatase inhibitor cocktail: sodium orthovanadate (10 mM), sodium fluoride (10 mM), and sodium pyrophosphate (10 mM). Brain tissues were homogenized by trituration and sonication. Tissue lysates were centrifuged at 13,500 RPM for 30 min at 4°C, and the supernatant was collected as a whole protein extract. Protein was quantified using the Pierce BCA protein assay kit (Thermo Scientific, Rockford, IL, USA). Equal amounts of protein were resolved on SDS-PAGE followed by transfer to polyvinylidene difluoride membranes (Millipore, Bedford, MA) using transfer buffer (25 mM Tris, 192 mM glycine, and 20% methanol). The membrane was subsequently blocked with 5% non-fat skim milk in TBST (Tris-buffered saline containing 0.1% vol/vol Tween-20) for 1 h at room temperature, followed by incubation in respective primary antibodies (Table 1) in blocking solution overnight at 4°C. Membranes were then subjected to washes in TBST, followed by incubation with HRP-conjugated secondary IgG. Blots were washed in TBST, and immunoreactive bands were visualized using SuperSignal^TM^ West Pico PLUS Chemiluminescent Substrate (Thermo Fisher Scientific). Densitometric analyses of non-saturated membranes were carried out using a Syngene G: Box chemidoc system and Image J software.

### Gene expression: RNA isolation, reverse transcription, and quantitative polymerase chain reaction

RNA was extracted from the whole brain or spinal cord tissues (flash-frozen) of MHV-A59, MHV-A59+4-PBA, and mock-infected mice using the Trizol isolation protocol following transcardial perfusion with DEPC-treated PBS. The total RNA concentration was measured using a NanoDrop ND-2000 spectrophotometer. 1 µg of RNA was used to prepare cDNA using a High-Capacity cDNA Reverse Transcription Kit (Applied Biosystems). Quantitative Real-time PCR analysis was performed using iTaq UniverSYBR Green qPCR kit (BioRad) in a BioRad CFX Real-time PCR system (BioRad) under the following conditions: initial denaturation at 95°C for 7 min, 40 cycles of 95°C for 10 s, 60°C for 30 s, and melting curve analysis at 60°C for 30 s. Reactions were performed in quadruplets. Relative quantitation was performed using the comparative threshold (ΔΔCt) method. mRNA expression levels of target genes in MHV-A59, MHV-A59+4-PBA, and mock-infected mice were normalized with GAPDH and expressed as relative fold change compared with their respective mock-infected controls. Primer sequences are listed in Table 2.

**TABLE 2:** Sequences of primers used in the study

Primer	Forward primer	Reverse primer
Cx43	CCCTTCACGCGATCCTTA	TCATGCTGGTGGTGTCCTTG
N-gene	GTTGCAAACAGCCAAGCG	GGGCGCAAACCTAGT
ERp29	GGCAGTTAAGGTTGGAGCCATCCAG	TATGCTGGAGGCCTTGATGAACTCGC
TNF α	CTGTAGCCCACGTCGTAGC	TTGAGATCCATGCCGTTG
IL-1 β	TGTAATGAAAGACGGCACACC	TCTTCTTTGGGTATTGCTTGG
IFN γ	GTCTCTTCTTGGATATCTGGAGGAACT	GTAGTAATCAGGTGTGATTCAATGACGC
IL6	AGTTGCCTTCTTGGGACTGA	TCCACGATTTCCCAGAGAAC
Cx47	AGAACTTGGCGAACCTAGCG	AGAGGTTGCATTGAGCCCAG
TGF-β	CAAGGGCTACCATGCCAACT	GTACTGTGTGTCCAGGCTCCAA
IFN α	CTTCCACAGGATCACTGTGTACCT	TTCTGCTCTGACCACCTCCC
IFN β	CTGGCTTCCATCATGAACAA	AGAGGGCTGTGGTGGAGAA
Ifit2	GGGAAAGCAGAGGAAATCAA	TGAAAGTTGCCATACAGAAG

### Immunofluorescence microscopy

Brain tissues were harvested and embedded in paraffin following transcardial perfusion with PBS. Tissues were post-fixed in 4% paraformaldehyde for 36–48 h, following which tissues were processed in increasing concentrations of ethyl alcohol, xylene, and paraffin wax, embedded in paraffin, and sectioned into 5-µm thick mid-sagittal sections of the brain containing olfactory bulb, cerebral cortex, hippocampus, hypothalamus, midbrain, cerebellum, and part of brain stem using the Thermo Scientific HM 355 S sectioning system. The serial tissue sections parallel to those used for corresponding immunohistochemistry were stained. Briefly, the slides were deparaffinized, followed by rehydration and antigen unmasking. The slides were then permeabilized with 0.2% Triton X-100 in PBS by shaking incubation at RT for 15 min and blocked using 1% bovine serum albumin (BSA) prepared in 0.2% Triton X-100 in PBS at 37°C for 1 h. This was followed by shaking incubation at 4°C for 16 h with primary antibodies prepared in blocking solution. Each section on each sample slide was stained for the following separately: (i) anti-GFAP, (ii) anti-Iba1, (iii) anti-viral nucleocapsid, (iv) anti-Cx43 and anti-viral nucleocapsid, (v) anti-ERp29 and anti-viral nucleocapsid, (vi) and anti-Cx47 and anti-viral nucleocapsid. Slides were then washed and incubated for 1 h at 37°C with a combination of secondary antibodies AlexaFluor568 and/or AlexaFluor488 prepared in blocking solution. Finally, the slides were washed in PBS and mounted using Vectashield with DAPI.

Images were acquired using a Spinning Disk (Confocal, SORA) Microscope using a 10× objective for Fig. 4 to 6 and using the Leica SP8 confocal platform using an oil immersion 63× objective (NA 1.4) and deconvolved using Leica Lightning software for Fig. 9 and 10. The images were captured at a z-interval of 0.4 µm for Fig. 9 and 10.

### Gap 27 peptide treatment

The Cx43 mimetic peptide Gap 27 (SRPTEKTIFII, amino acids 201–211 from the second extracellular loop of Cx43) was purchased from Tocris. The mechanism of action of Gap 27 mimetic peptide has been extensively studied (27, 57). The Gap 27 peptide binds to the Cx43 extracellular loop domain 2 and closes Cx43 hemichannels within minutes, where the disruption of gap junction-mediated cell coupling occurs at later time points. The primary astrocytes were pre-treated with 300 µM Gap 27 or maintained in normal growth medium without any mimetic peptide for 24 h prior to infection with MHV-A59, which is known to block Cx43–mediated GJIC (25). Immunofluorescence for viral N was carried out at 24 h post-infection, followed by calculation of the percentage of infected cells.

### Statistical analysis

Values were represented as mean values  ±  standard errors of the mean (SEM). Values were subjected to unpaired Student’s *t*-tests with Welch’s correction or ordinary one-way ANOVA with multiple comparison tests (Tukey’s test and the Holm-Sidak test) for calculating the significance of differences between the means. All statistical analyses were performed using GraphPad Prism 8 software (La Jolla, CA). A *P*-value of  < 0.05 was considered statistically significant.

## Data Availability

The data supporting the study findings can be obtained from the corresponding author on request.
